# The Complete Genome of an Endogenous Nimavirus (*Nimav-1_LVa*) From the Pacific Whiteleg Shrimp *Penaeus* (*Litopenaeus*) *Vannamei*

**DOI:** 10.3390/genes11010094

**Published:** 2020-01-14

**Authors:** Weidong Bao, Kathy F. J. Tang, Acacia Alcivar-Warren

**Affiliations:** 1 Genetic Information Research Institute, 20380 Town Center Lane, Suite 240, Cupertino, CA 95014, USA; 2 Yellow Sea Fisheries Research Institute, Chinese Academy of Fishery Sciences, 106 Nanjing Road, Qingdao 266071, China; 3 Fundación para la Conservation de la Biodiversidad Acuática y Terrestre (FUCOBI), Quito EC1701, Ecuador; 4 Environmental Genomics Inc., ONE HEALTH Epigenomics Educational Initiative, P.O. Box 196, Southborough, MA 01772, USA

**Keywords:** WSSV, white spot syndrome virus, *Nimaviridae*, *Nimav-1_LVa*, *DNAV-1_LVa*, *Penaeus* (*Litopenaeus*) *vannamei*

## Abstract

White spot syndrome virus (WSSV), the lone virus of the genus *Whispovirus* under the family *Nimaviridae*, is one of the most devastating viruses affecting the shrimp farming industry. Knowledge about this virus, in particular, its evolution history, has been limited, partly due to its large genome and the lack of other closely related free-living viruses for comparative studies. In this study, we reconstructed a full-length endogenous nimavirus consensus genome, *Nimav-1_LVa* (279,905 bp), in the genome sequence of *Penaeus* (*Litopenaeus*) *vannamei* breed Kehai No. 1 (ASM378908v1). This endogenous virus seemed to insert exclusively into the telomeric pentanucleotide microsatellite (TAACC/GGTTA)_n_. It encoded 117 putative genes, with some containing introns, such as *g012* (inhibitor of apoptosis, IAP), *g046* (crustacean hyperglycemic hormone, CHH), *g155* (innexin), *g158* (Bax inhibitor 1 like). More than a dozen *Nimav-1_LVa* genes are involved in the pathogen-host interactions. We hypothesized that *g046*, *g155*, *g158,* and *g227* (semaphorin 1A like) were recruited host genes for their roles in immune regulation. Sequence analysis indicated that a total of 43 WSSV genes belonged to the ancestral/core nimavirus gene set, including four genes reported in this study: wsv112 (dUTPase), wsv206, wsv226, and wsv308 (nucleocapsid protein). The availability of the *Nimav-1_LVa* sequence would help understand the genetic diversity, epidemiology, evolution, and virulence of WSSV.

## 1. Introduction

The pacific whiteleg shrimp *Penaeus* (*Litopenaeus*) *vannamei* is one of the most important penaeid species in the aquaculture and fishing industry. The natural range of wild *P. vannamei* populations is the pacific coast of Latin America, from northern Peru to northern Mexico. However, *P. vannamei* has been introduced into most of the shrimp-producing countries around the world, partly due to the domestication and availability of specific pathogen-free (SPF) stocks [1,2,3]. The term SPF means “healthy”, i.e., conditionally free of a list of known shrimp pathogens of the office of international epizootics (OIE), but not necessarily resistant and/or tolerant to any of the pathogens [3]. The first SPF *P. vannamei* was produced in Hawaii by the breeding program of the United States Marine Shrimp Farming Program (USMSFP) consortium and was maintained at the Oceanic Institute in Hawaii, USA [1,2]. Recently, the shrimp genome from the Kona line of the USMSFP was partially sequenced for a total length of ~470 Mb [1], from which numerous transposable elements, integrated viruses, and simple sequence repeats (SSRs) have been categorized [4] and deposited in Repbase [5]. Kona line is also known as research line, high-growth line, and/or Taura Syndrome Virus (TSV)-susceptible line, and was distributed to private commercial breeding companies [1]. In parallel, the genome of a male *P. vannamei* farmed in China (breed Kehai No. 1) was completely sequenced and assembled to be 1.66 Gb in size [6]. Although the expected genome size of *P. vannamei* ranges from 2.45 to 2.89 Gb [1], this 1.66 Gb scaffold sequence, in which 25,596 protein-coding genes were identified, would allow researchers to (a) complete a continuous whole-genome assembly of this highly complex species that contains the highest percentage of SSRs than any other species sequenced so far [1,6], (b) perform more basic epidemiology and evolutionary biology research, and (c) develop treatments and diagnostics tools for diseases of bacterial [1,7] and viral origin [8,9,10].

White spot disease (WSD) is the most devastating infectious shrimp disease. Infected shrimps are characterized by white spots (calcified deposits) on the exoskeleton. The first reported appearances of WSD in penaeid shrimp occurred in China (Fujian) in 1992 [11] and spread globally [10,12,13,14,15] to Taiwan, Korea, and Japan (1993), South East Asian countries (1996), United States (Texas and SC in 1995), India (1998), Latin America (1999), Madagascar, Mozambique and Saudi Arabia (2010–2012), and Australia (2016). The cause of WSD is large, enveloped dsDNA virus called white spot syndrome virus (WSSV) [16,17,18] that infects over 90 arthropod species naturally or experimentally [17,19], such as crayfishes, lobsters, crabs, and others. So far, 14 complete WSSV genomes of different isolates have been stored in GenBank, ranging between 280 Kb and 309 Kb in size, and are predicted to have ~180 open reading frames (ORFs) of 50 amino acids or above [16,18]. Different WSSV genomes share >95.22% overall sequence identity and could cluster in three or more phylogenetic groups [20,21]. In the Genbank database, many shrimp expressed sequence tags (ESTs) have been found showing homology to WSSV, especially when ESTs are from the SPF *P. vannamei* of the USMSFP breeding program from Hawaii [1]. WSSV fragments have been reported endogenized or integrated into an SPF stock of giant tiger shrimp (*Penaeus monodon*) from Thailand [22], showing Mendelian inheritance [23]. A recent study in Kuruma shrimp, *Penaeus* (*Marsupenaeus*) *japonicus*, illustrated that the entry of WSSV into the host cell is via the endocytosis pathway, triggered by the interaction of virion and a transmembrane immunoglobulin receptor, designated as *Mj*pIgR [24]. So far, progress has been made in developing WSSV-resistant *P. vannamei* lines [25,26], but a lot more work remains ahead to achieve the stabilization of the resistance.

WSSV has long been regarded as the lone virus (type species) of the genus *Whispovirus*, which is the only genus of the family *Nimaviridae* [18]. However, this notion is changing with the recent discovery of diverse endogenous WSSV-like nimaviruses [27,28,29,30]. In some crustacean genomes, such as *P. monodon* (Pm), even two different types of endogenous nimaviruses can be distinguished [28]. The genome scaffolds of these endogenous nimaviruses vary in length from ~190 Kb to ~230 Kb, but none is considered a complete virus genome [28]. According to the phylogeny reported by Kawato et al. [28], family *Nimaviridae* currently consists of seven major phylogenetic groups (or genus, if diversity qualified), and different groups share less than 60% DNA sequence identity to each other [28]. The representative viruses of the seven groups are WSSV, *Chionoecetes opilio bacilliform virus* (CoBV), and the five endogenous nimaviruses from *Penaeus* (*Marsupenaeus*) *japonicus* (Mj), *Penaeus monodon*, *Hemigrapsus takanoi*, *Metapenaeus ensis*, *Sesarmops intermedium*, respectively (Table 1). Comparative analysis showed that 39 WSSV genes could be termed as ancestral/core nimavirus genes since their orthologs were ubiquitously (core) or widely (ancestral) present in the seven *Nimaviridae* lineages, particularly in the Mj nimavirus, which belongs to the most distant group (Mj-group) to WSSV [28]. These 39 genes include envelope proteins, capsid proteins, DNA polymerase, protein kinase, and some other hypothetical or unknown proteins. In other words, these ancestral/core genes (families) are rarely lost in the course of evolution [31].

From the ~470 Mb genome of the first SPF *P. vannamei* [1], we previously reconstructed a 279,384 bp long consensus sequence, designated as *DNAV-1_LVa*, to represent the complete genome of a WSSV-like virus [29,30]. In Repbase [5], *DNAV-1_LVa* is stored as seven smaller segments (entries): *DNAV-1a_LVa* to *DNAV-1g_LVa*. We reported here an updated version of this WSSV-like nimavirus, reconstructed from the high-quality sequence data of *P. vannamei* Kehai No. 1 genome [6]. This new consensus was designated as *Nimav-1_LVa* (279,905 bp) to emphasize its upgraded quality over *DNAV-1_LVa*. With about 65–74% sequence identity to the Mj endogenous nimavirus, *Nimav-1_LVa* clearly belonged to the Mj-group. In *Nimav-1_LVa*, 117 protein-coding genes were predicted, including four genes newly demonstrated as nimavirus ancestral/core genes. In addition, four other *Nimav-1_LVa* genes might be captured host genes for their regulatory roles in the host-pathogen interactions and/or immune response. This complete genome of *Nimav-1_LVa* might provide a useful source to aid in our understanding of the evolution of virus family *Nimaviridae*.

## 2. Materials and Methods

### 2.1. Nimav-1_LVa Virus Consensus Reconstruction

The process of reconstructing the consensus of various repetitive families have been described elsewhere [5]. Briefly, RepeatModeler [32] tool was used to initially identify “pre-consensus” sequences in the genome. These “pre-consensus” sequences were used by BlastN to bait out top hit sequences in the genome, from which the consensus sequences were reconstructed again. To extend to the complete length of a given family, a stepwise extension in both directions was performed until the sign of termini appears. The consensus of *Nimav-1_LVa* is provided in Supplementary File S1.

### 2.2. Viral Gene Prediction and Visualization

*Nimav-1_LVa* genes or ORFs were predicted in three steps. First, ORFs with 70 codons or above were predicted. ORFs completely overlapped by other larger ORFs or that largely derived from simple sequences or tandem repeats were discarded. The tandem repeat region was predicted by Tandem Repeat Finder [33] (TRF, Version 4.09) with default parameters. Second, regions consisting of multiple adjacent short ORFs in the same direction were subjected to online FGENESH [34] prediction to check the possibility of exon-containing genes. We chose *Apis dorsata* (giant honey bee) as the species parameter for FGENESH since the predicted proteins proved more correct than using some other species. Lastly, to further reduce the error in gene prediction, the predicted proteins were subjected to comparative TblastN or BlastP analyses against either the *Nimav-1_LVa* or the other nimaviruses. By this approach, we corrected a few frameshifts caused by ambiguity in short tandem repeats. Some obvious duplicated partial gene fragments were also discarded. The 117 protein sequences of *Nimav-1_LVa* are provided in Supplementary File S1. Multiple sequence alignment (MSA) was performed by an online MAFFT server [35] and was visualized in Jalview [36].

### 2.3. Homology Searches

Protein homology searching (TblastN or BlastP) was performed locally with the Censor tool [37] implemented with Wu-blast (version 2.0) search engine. Protein database searching was conducted by BlastP or PSI-Blast (Position-Specific Iterated Blast) at NCBI (https://blast.ncbi.nlm.nih.gov/Blast.cgi?PAGE=Proteins). HMMER3 [38] software was used to detect more distant viral proteins. MSA alignment was constructed using online MAFFT [35], version 7.423, and HMM (hidden Markov models) profile generated were used in HMMSEARCH in the HMMER3 suite.

### 2.4. Dataset

Nimavirus genomes or assemblies used in this paper for comparative analysis included 3 WSSV genomes (AF332093.3, WSSV-CN; AF369029.2, WSSV-TH; and KT995472.1, WSSV-CN01), *Metopaulias depressus* (Md, KR820240 to KR820242), and the other 6 genomes listed in Table 1. Except for the 3 WSSV genomes, all other nimaviruses genomes were incomplete. The whole-genome sequences (WGS) of *Penaeus monodon* isolate Shenzhen (NIUS000000000) and *Marsupenaeus* (*Penaeus*) *japonicus* isolate Guangxi (NIUR010000000) were downloaded from GenBank.

## 3. Results

### 3.1. Building the Consensus of Nimav-1_LVa

Using the PacBio sequencing method, we previously conducted a small-scale genome sequencing project on the SPF *P. vannamei* Kona line of the USMSFP [1]. Around 470 Mb sequences were randomly obtained from the genome. From this data, a 279,384 bp long WSSV-like consensus sequence was reconstructed and was deposited in Repbase [5] under the name *DNAV-1_LVa* [29]. Due to the high error rate of PacBio sequencing, and the low genome coverage of the data, the sequence quality of *DNAV-1_LVa* proved prohibitive for a thorough analysis. In this study, we reconstructed this *DNAV-1_LVa*-like consensus using the high-quality genome sequences of *P. vannamei* breed Kehai No. 1 variety (GenBank assembly No. ASM378908v1) that were generated by both PacBio and Illumina platforms. We designated the new consensus with a different name: *Nimav-1_LVa*, to reflect its being a nimavirus and emphasize its superior sequence quality to the original *DNAV-1_LVa*. *Nimav-1_LVa* was 279,905 bp long, ~98% identical to *DNAV-1_LVa* sequence, and showed the same overall structure, but length variations were observed in some tandem repeat regions. The sequence of *Nimav-1_LVa* is provided in Supplementary File S1.

**Figure 1:**
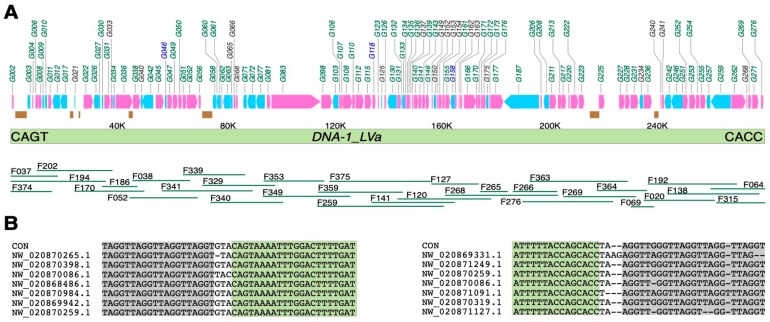
(**A**) Schematic representation of *Nimav-1_LVa* endogenous nimavirus and the encoded genes. Uppermost part indicates the location of genes and their transcription orientation (in pink or light blue). Gene names in black are 20 hypothetical genes, gene names in blue (*n* = 3) indicate no viral homologs are found, genes in green (*n* = 94) indicate viral homologs are found. The brown boxes indicate the locations of long tracts of simple tandem repeats. Solid lines below the *Nimav-1_LVa* bar represent some larger *Nimav-1_LVa* segments present in the Kehai No. 1 assembly. The accession number and location of the segments can be found in Supplementary Table S1; (**B**) Sequence alignment of the terminal regions of the linear *Nimav-1_LVa* and the flanking sequences. The green-shaded regions belong to *Nimav-1_LVa*. The telomeric (TAACC/GGTTA)_n_ microsatellite regions are shaded in grey.

In the *Nimav-1_LVa* sequence, except for a ~1.8 Kb region (184,126 to 185,979 nt) and its immediate ~100 bp flanking sequences, the whole *Nimav-1_LVa* consensus was well-supported by at least three long genomic sequences from different loci (Figure 1A), all >98% identical to the consensus. In the current shrimp Kehai No. 1 genome assembly, this 1.8 Kb sequence occurred only in one contig NW_020871279.1. In another contig NW_020871249.1 from the same genomic locus, this 1.8 Kb region was substituted by a 413-bp unsequenced polyN tract (491,007–491,419 nt). Luckily, this 1.8 Kb region was located within the coding region of the gene *g187* (Figure 1A), which encoded in its single, long ORF a 4332 AA protein (187p), showing 56% identity over the whole length to a wsv343-like protein BBD20111.1 (4287 AA) encoded in Mj nimavirus. Thus, this poorly-supported 1.8 Kb region would not seriously affect our subsequent analysis.

In the current 1.66 Gb genome assembly of the shrimp breed Kehai No. 1, a total of 3335 Kb sequences was found to be derived from *Nimav-1_LVa*: >95% identity to the consensus, and 80% of these sequences showed >98% identity to the consensus (Supplementary Table S1). These data indicated that at least 12 copies (3335/279 = 11.9) of *Nimav-1_LVa* were integrated into the shrimp genome during the relatively recent past. Among the available endogenous nimaviruses assemblies, *M. japonicus* (Mj) endogenous nimavirus (BFCD01000001 and AP010878) [28] was the closest relative to *Nimav-1_LVa*. They shared a 65–74% nucleic acid sequence identity to each other, and both featured low GC-content: 34.6% in *Nimav-1_LVa* and 32.9% in the Mj endogenous nimavirus. By contrast, all other nimaviruses genomes exhibited significantly high GC-content: 45% in the Pm endogenous nimavirus, 47% in the Ht endogenous nimavirus, 45.4% in the Me endogenous nimavirus, 44.2% in the Si endogenous nimavirus, 44.1% in the Md endogenous nimavirus, 41% in WSSV, and 40% in the *Chionoecetes opilio bacilliform virus*.

### 3.2. The Integration Site of Nimav-1_LVa

As shown in Figure 1A, the integration site on the circular virus genome was located between gene *g002* and gene *g276*. Hereafter, the orientation of the linear *Nimav-1_LVa* was defined as in Figure 1A. In the assembly of shrimp breed Kehai No. 1, a total of 21 genomic loci were juxtaposed with the termini of *Nimav-1_LVa*: 10 loci at the 5′-end and 11 at the 3′-end. The number of these termini (21) accorded well with the number of the integrated *Nimav-1_LVa* copies (12), which was deduced from the total length of the viral sequences. Thus, this data implied that the site between *g002* and *g276* was the only possible recombination site on the virus genome. Moreover, we found all these *Nimav-1_LVa* copies were flanked by a long tract of (TAACC/GGTTA)_n_ microsatellites (Figure 1B), which were reported as the telomeric sequence in *P. vannamei* [6,39]. Notably, the (TAACC/GGTTA)_n_ microsatellite region was internally absent in the *Nimav-1_LVa* consensus, strongly indicating that the integration between *Nimav-1_LVa* and the host genome happens preferentially, if not exclusively, between one specific virus site and the telomeric microsatellite repeats. However, one caveat must be noted that the *Nimav-1_LVa* might also integrate into non-telomeric regions, but these viruses had been subsequently eliminated during evolution.

The precise boundary between integrated *Nimav-1_LVa* and shrimp genome is undetermined yet. The termini of this linear *Nimav-1_LVa*, 5′-CAG, and ACC-3′, as illustrated in Figure 1, were approximate and tentative. No obvious target site duplications (TSDs) were observed flanking *Nimav-LVa*. Little is known about the molecular mechanism underlying such integration because we cannot exclude the possibility that circular *Nimav-1_LVa* could harbor one short tract of variable length of (TAACC/GGTTA)_n_ microsatellites somewhere between *g002* and *g276*. If so, the integration of *Nimav-1_LVa* would be through the homology-based recombination, which is adopted in the telomere-specific integration of human herpesvirus HHV-6A, HHV-6B [40,41,42], and chicken lymphotropic alphaherpesvirus Marek’s disease virus (MDV) [43,44].

### 3.3. Nimav-1_LVa Sequences in Other Penaeid Shrimps

To test if *Nimav-1_LVa* is present in other shrimp species, we blasted the *Nimav-1_LVa* sequence against the two available whole-genome sequences (WGS) of *P. monodon* isolate Shenzhen (NIUS000000000, 1.4 Gb) and *M. japonicus* isolate Guangxi (NIUR010000000, 1.6 Gb). In addition, we performed two similar searches using the Mj-type and the Pm-type endogenous nimaviruses (Table 1). As a result, a substantial amount of homologous sequences, either identical (>99%) or highly homologous (>88%), was detected in the two genomes. The detected homologous viral sequences seemed to scatter throughout the whole virus genome; in some specific locations, even three different versions of viral sequences could be detected. The cumulative lengths of the homologous sequences in each search are listed in Table 2. The varying amounts of the integrated viral sequences might be accounted for by the different magnitudes of infection and different levels of host tolerances to the integration of different viruses. These data suggested that at least three types of nimavirus sequences were integrated into the two shrimp isolates from *P. monodon* and *M. japonicus*. The first virus type was obviously the *Nimav-1_LVa* type (>99% identity). The other two types, given the fairly high sequence identity (>88% or >91%) to the query sequences, could be called Pm-like and Mj-like (Table 2). Putting together, the identification of almost identical *Nimav-1_LVa* sequence in three species, *P. monodon*, *M. japonicas,* and *P. vannamei* (previous section), highly suggested that *Nimav-1_LVa* virus or its closest variant is or was a potentially transmissible virus in nature.

### 3.4. Genes Encoded in Nimav-1_LVa

In the *Nimav-1_LVa* sequence, a total of 117 protein-coding genes were predicted (Table 3 and Supplementary File S1 for the protein sequences), each with 70 codons or longer. Ninety-seven of the genes were supported by homologous proteins, mostly from other nimaviruses (Table 3). The remaining 20 genes were hypothetical, generally short, with the exception of only two genes (*g153* and *g234*) coding for proteins over 400 residues.

Twenty-eight out of the 117 genes were found homologous to at least one other *Nimav-1_LVa* gene. Based on their mutual similarity, these genes were clustered into six “paralog families” (PF): PF1 (*g002*, *g006*, *g008*, *g009*, *g010*, *g011*, *g141*, *g143*, *g146*, *g161*), PF2 (*g003*, *g012*, *g017*, *g030*, *g047*, *g049*), PF3 (*g050*, *g051*, *g052*, *g257*), PF4 (*g172*, *g173*, *g276*), PF5 (*g056*, *g269*, *g271*), and PF6 (*g034 and g139*). Notably, it was possible that in some gene families, some shorter genes were just pseudogenes or gene fragments due to partial duplication or to the errors in gene prediction, such as the *g002* gene in the PF1 family, the *g030* in the PF2 family (Table 3). In the PF3 family, *g052* was much longer than the rest of the members, and the homologous region was limited to the N-terminal half region of *g052*. Nevertheless, for the purposes of documentation, these genes are still enlisted in Table 3.

PF1 was the largest gene family with a total of 10 family members, reflecting its critical roles for the virus. However, the roles of PF1 families were largely unknown: no significant conserved domain was found. In the PF2 family, all six members contained one to three BIR domains (baculoviral inhibition of apoptosis protein repeat, cd00022) (Table 3). In addition, a carboxyl-terminal zinc-finger domain of the RING-HC (C3HC4-type) subclass was present in four PF2 members. The four zinc-finger domains belonged to two subtypes: RING-HC_BIRC2_3_7 (cd16713) in *g012* and *g017*, and RING-HC_BIRC4_8 (cd16714) in *g047* and *g049* (Table 3). The BIR and RING domain arrangement is also found in a number of well-studied inhibitors of apoptosis (IAP) proteins [45]. As indicated by the acronym BIRC (baculoviral IAP repeat-containing protein) in the zinc-finger subtype name, the other IAP proteins include BIRC2 (also known as c-IAP1, cellular inhibitor of apoptosis protein 1), BIRC3 (c-IAP2), BIRC7 (Livin), BIRC4 (XIAP, X-linked inhibitor of apoptosis protein), and BIRC8 (ILP-2, IAP-like protein 2). It is known that these IAP proteins act as ubiquitin E3 ligases to mediate the ubiquitination of the substrates involved in apoptosis, nuclear factor-kappaB (NF-kappaB) signaling, and oncogenesis [46]. BIRC3 influences ubiquitin-dependent pathways that modulate innate immune signaling by activation of NF-kappaB, and BIRC4, 7, 8 are all implicated in the effect of anti-apoptosis [45,46,47].

One striking feature of *Nimav-1_LVa* was that exon-intron structures are found in nine genes, including five PF2 family genes (*g003*, *g012*, *g017*, *g047,* and *g049*), *g022*, *g046* (CHH), *g155* (innexin), and *g158* (BAX inhibitor 1-like) (Table 3). While the exons in *g022* have yet to be confirmed by other independent resources, the existence of exons seemed to be positively confirmed for the other eight genes by their homologs from GenBank. Notably, no WSSV gene is found to be spliced so far [18].

It has been known that 39 WSSV genes and their homologs are commonly present in nimaviruses, in particular, Mj-type nimavirus and WSSV [28], and are so-called nimavirus ancestral/core genes. However, because of the incompleteness of the current scaffold of the Mj-type nimavirus genome (~220 Kb, Table 1), this ancestral/core gene set could be incomplete. Given the close relationship between *Nimav-1_LVa* and *M. japonicus* (Mj) nimavirus, both under the Mj-group [28], we examined the possible homologous genes between *Nimav-1_LVa* and WSSV, aiming at additional *Nimav-1_LVa* genes that could be included into the ancestral/core gene set.

**Figure 2:**
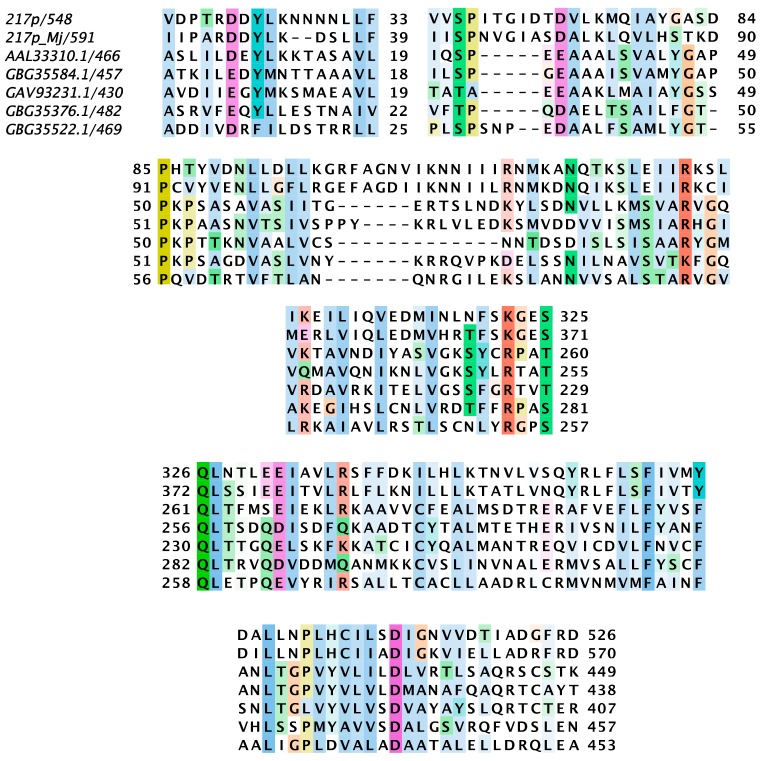
Conserved blocks in the alignment of 217p, wsv308 (AAL33310.1), and other homologs. 217p is the protein encoded by gene *g217* in *Nimav-1_LVa*, 217p_Mj denote the homolog of 217p encoded in Mj nimavairus. The other proteins and the hosting genome are AAL33310.1 (WSSV), GBG35584.1 (Si nimavirus), GAV93231.1 (*Chionoecetes opilio bacilliform virus*), GBG35376.1 (Ht nimavirus), and GBG35522.1 (Pm nimavirus). Numbers after the slash indicate the length of that protein. Numbers at either side of the blocks indicate the locations of the preceding/following amino acids in each protein.

As a result, 44 *Nimav-1_LVa* genes were found homologous to 43 WSSV genes. These paired homologous genes are indicated with “wsvNNN-like” in the “Comment” column in Table 3. The WSSV genes here referred to those annotated for the genome of the WSSV CN strain (AF332093.3). Of the 44 *Nimav-1_LVa* genes, 39 genes proved to be the orthologs of the known 39 ancestral/core genes [28], the other five newly-included genes were *g140* (wsv112-like), *g217* (wsv308-like), *g225* (wsv226-like), *g034* (wsv206-like), and *g139* (wsv206-like). The last two genes were two paralogs belonging to the PF6 gene family. These five newly identified proteins showed marginal similarity (<30% amino acids identity), or no detectable similarity, to their WSSV counterparts by BlastP; however, their orthology was well-supported in the multiple sequences alignment (MSA) (Figure 2 and Supplementary Figures S1–S3). For example, although the *g217*-encoded protein (217p) showed no detectable similarity with the wsv308 protein, also called VP51, a nucleocapsid protein [48], it did show trace similarity (<18% identity) with another *S. intermedium* (Si) nimavirus protein GBG35584.1, which was annotated as a wsv308-like protein [28]. When 217p, GBG35584.1, wsv308, and some other wsv308-like proteins were included in the multiple sequence alignment, the orthology was clearly revealed by the many highly-conserved residues/blocks throughout the whole length (Figure 2). Similarly, we concluded that *g140* was a wsv112-like dUTPase enzyme (Supplementary Figure S1); *g225* was wsv226-like (Supplementary Figure S2); and the two PF6 members, *g034* and *g139*, as well as their homologs in Mj nimavirus (GBG35398.1 and GBG35402.1), were indeed homologs of wsv206 (Supplementary Figure S3). Admittedly, Kawato et al. did acknowledge that GBG35398.1 and GBG35402.1 were likely homologs of wsv206, but this uncertainty was unsolved in the paper [28]. Notably, the wsv206-like protein GBG35398.1 contains a macro domain (cl00019, E-Value = 3.00076 × 10^−5^), which is a high-affinity ADP-ribose binding module.

Besides the 44 ancestral/core genes, eight *Nimav-1_LVa* genes were found with equivalents in the non-WSSV and non-Mj-group nimaviruses. The absence of WSSV homologs for these genes could be explained by the gene loss in WSSV. The eight genes included *g115*, *g206,* and the six inhibitors of apoptosis from the PF2 family. The counterparts of *g115* (SCV_095, GAV93215.1) and *g206* (SCV_028, GAV93152.1) were encoded in CoBV. The BIR domain in the PF2 family members was absent in WSSV proteins, but it was encoded in one Md nimavirus protein (AKS10635.1), one CoBV protein (GAV93213.1), and one Ht nimavirus protein (GBG35369.1).

The remaining 45 homolog-supported genes could only find their homologs from the Mj-type nimavirus or the non-redundant (nr) protein database of NCBI. These 45 genes and the 20 hypothetical genes were tentatively called “Mj-group-specific” genes (indicated in bold font, Table 3). Theoretically, these “Mj-group-specific” genes comprised three sections: (1) genes that were acquired in the common ancestor of the Mj-group after its split from other nimaviruses, (2) genes whose orthologs have been lost in the evolution of other nimaviruses, (3) genes underwent faster evolutionary rate, thus making it difficult to detect their homologs in other virus groups. Unless more nimavirus genomes are completely assembled, a lot of uncertainty remains in this area.

### 3.5. Nimav-1_LVa Genes Involved in Host-Pathogen Interaction

Although the molecular functions of a lot of *Nimav-1_LVa* proteins were unknown, a large number of genes/families seemed connected to roles in host-pathogen interaction and innate immune response. These genes/families included: (1) *g103* (heat shock protein, Hsp70), (2) *g118* (DnaJ, also called Hsp40), (3) *g132* (ubiquitin), (4) the 6 IAPs of the PF2 family, (5) *g171* (wsv267-like anti-apoptotic protein), (6) *g046* (CHH), (7) *g155* (innexin), (8) *g158* (BAX inhibitor 1 like), and (9) *g227* (semaphorin 1A like).

In the cases of the first four genes/families: *g103* (heat shock protein, Hsp70), *g118* (Hsp40), *g132* (ubiquitin), and the six inhibitors of apoptosis of the PF2 gene family, their involvements in the host-pathogen interaction were well acknowledged. It is well known that apoptosis is a key immune process in the shrimp response to the WSSV invasion [49]. Various heat shock proteins and ubiquitin are also well documented for their functions in host-virus interaction. For example, extracellular Hsp70s have been demonstrated with a number of cytoprotective and immunomodulatory functions, such as stimulators of innate immune responses in the human system [50]. A heat shock protein 70 (Hsc70) was found to inhibit apoptosis induced by WSSV infection in hemocyte shrimp cells [51]. In shrimp *P. vannamei*, the expression of the Hsp70 gene was also reported altered after the WSSV infection [52,53], and intramuscularly injection of Hsp70 protein could significantly reduce mortality after WSSV infection [54]. As for the Hsp40 gene, its responses to viral infection have been reported in halibut *Paralichthys olivaceus* [55]. In another study using the WSSV challenged tiger shrimp *P. monodon*, ubiquitin gene was down-regulated during the first 12 hours, but reversed in the following period [56]. Lastly, a study in red swamp crayfish, *Procambarus clarkii*, listed DnaJ (Hsp40), ubiquitin, and innexin (detailed below) proteins for their possible anti-WSSV roles [57].

The *g171* gene is the ortholog of WSSV wsv267. The wsv267 protein, also known as anti-apoptotic protein 4 (APP4) [18], has been shown capable of inhibiting apoptosis by binding with the p20 domain of *P. monodon* caspase (PmCasp) protein, which can induce apoptosis [58]. There are four anti-apoptotic WSSV proteins identified (APP1 to APP4) [18], but only APP4 (wsv267) protein could find its homolog in *Nimav-1_LVa* (171p).

In the cases of the last four genes, *g046* (CHH), *g155* (innexin), *g158* (BAX inhibitor 1 like), *g227* (semaphorin 1A like), their roles in virus infection was not obvious. The *Nimav-1_LVa g046* gene encodes a 123 AA protein (ROT61446), which is 59% homologous to the crustacean hyperglycemic hormone (CHH) like protein encoded by gene KJ660843 [59]. Both proteins are encoded in three coding-exons and are co-classified in the CHH group named as type-Ib [6]. Notably, there are around 21 type-Ib CHH genes in the *P. vannamei* genome [6], and 13 of them seem to be accounted for by this viral *g046*. In addition to the manifold functions in blood glucose regulation, control of the molt cycle, osmoregulation, etc. [60,61], CHH peptides can increase the survival rate of bacteria-infected shrimp [62] and might be involved in hemocyte intracellular signaling pathways to regulate exocytosis and immune response [63].

Gene *g155* encodes a membrane protein innexin (pfam00876), which is functionally analogous to the vertebrate connexin in the cell gap junction [64,65]. There are 21 innexin genes in *P. vannamei* [6], and some of them are due to the multiplication of the viral genome. Innexin is involved in immune response and cell apoptosis [65,66], probably by regulating the closure of the gap channel to reduce the neighboring cellular apoptosis [67,68]. In a study in red swamp crayfish, the innexin gene has been listed as a candidate anti-WSSV gene [57]. Notably, the *g155* gene contains four exons, and its homolog is found in Mj nimavirus (BFCD01000001.1). Interestingly, innexin-like genes were also reported in a number of parasitoid viruses from the *Ichnovirus* genus in the *Polydnaviridae* family, such as *Campoletis sonorensis* ichnovirus (CsIV) and *Hyposoter didymator* ichnovirus (HdIV) and *Hyposoter fugitivus* ichnovirus (HfIV) [69,70], where innexins are termed vinnexins but are viewed as orthologs of host innexins acquired by the viruses since they show strong sequence similarity to insect innexins [69,70]. However, unlike the *Nimav-1_LVa* encoded *g155* (innexin), these vinnexin genes lack introns [71].

Gene *g158* encodes a BAX inhibitor (BI)-1-like protein (cd10430), which is located primarily in the membranes of the endoplasmic reticulum (ER) and suppresses ER stress-induced apoptosis [72,73]. BI-1 is a conserved suppressor of programmed cell death in animals and plants [74]. Gene *g158* also contains exons, but its homolog is not found in the Mj nimavirus genome, probably due to the incompleteness of the current Mj nimavirus assembly. Interestingly, the genomic loci of g158 are next to that of *g155* (Table 3).

Gene *g227* encodes a trans-membrane semaphorin 1A (Sema1A)-like protein (Class 1 semaphorins). While semaphorins generally act as signaling ligands that regulate the shape and motility of cells, their roles in immunity have been noticed [75,76]. Membrane-associated semaphorins play a role in regulating immune homeostasis in mouse models [77], according to which CD72 (Cluster of Differentiation 72) and TIM-2 (T cell immunoglobulin and mucin domain protein 2) ligands functionally interact with semaphorin Sema4D and Sema4A, respectively [78]. Although direct evidence supporting the involvement of Sema1A in immune regulation still lack in invertebrate system, the finding of Sema1A-like protein encoded in a virus-like *Nimav-1_LVa* is probably not a simple coincidence, especially considering that the other three cellular-like genes, *g046* (CHH), *g155* (innexin), *g158* (BAX inhibitor 1 like), are all present in *Nimav-1_LVa*, likely involved in pathogen-host interactions. Therefore, we hypothesized that *g227* (semaphorin 1A like) could also have a potential role in immune regulation.

## 4. Discussion

### 4.1. Nimav-1_LVa Consensus Sequence

We reported reconstructing a 279 Kb long, high-quality consensus sequence from the genome of *P. vannamei* breed Kehai No. 1 variety farmed in China [6], to represent the complete genome of an endogenous nimavirus, *Nimav-1_LVa.* This consensus sequence showed a ~98% sequence identity to our previous *DNAV-1_LVa* consensus reconstructed from the first SPF *P. vannamei* domesticated in the US [1,30,39]. It was reported that Kehai No. 1 was derived from Hawaii, USA, as well [79]. However, it remains to be determined if the original Kehai No. 1 stocks were purchased from a private American shrimp breeding company (High Health Aquaculture, HHA) based in Kona, Hawaii, or from the original SPF Kona line of the breeding program of the USMSFP Consortium, which was funded by the USDA-CSREES and maintained at The Oceanic Institute in Honolulu, Oahu, Hawaii until 2009 [1]. This 279 Kb of *Nimav-1_LVa* is very close to the known genome size range of WSSV viruses (280–309 Kb) but much larger than the current scaffold assemblies of all other endogenous nimaviruses (Table 1). This is probably because only those contigs bearing homology to WSSV sequences are considered [28]. The successful reconstruction of *Nimav-1_LVa* is largely attributed to two factors that a large quantity of *Nimav-1_LVa* remnant is present in the shrimp genome and that the integration of *Nimav-1_LVa* is a relatively recent event. Given the high sequence similarity among the large *Nimav-1_LVa* fragments in the genome, the question arises of if this *Nimav-1_LVa*, coupled with the highly abundant (>23.93%) SSRs [6], could cause any assembling problem, and to what extent. With hindsight, the current 1.6 Gb Kehai No. 1 assembly is quite apart from the expected 2.45 to 2.89 Gb of *P. vannamei* [1]. Considering the complexity of the shrimp genome, it would be good to have another genome assembly from a different *P. vannamei* stock available in the future.

Compared to the sequence from individual loci, consensus sequence possesses the merit of restoring the viral sequence to its early state when the integration first happened, thus minimizing the adverse effects caused by numerous sequence mutations. Gene prediction made on the consensus would be more accurate. For instance, in the Mj nimavirus sequence (BFCD01000001.1), the corresponding coding region of *Nimav-1_LVa* gene *g130* (1145 AA) is interrupted by a frameshift mutation.

Sequence analysis in the shrimp genome indicates *Nimav-1_LVa* viruses integrate exclusively into telomeric microsatellite (TAACC/GGTTA)_n_ [6,39]. The telomere-specific integration pattern could be partly explained by negative selection on those integrations in the non-dormant regions because, as demonstrated in human herpesvirus 6A and 6B (HHV-6A and HHV-6B) [41], insertion into telomere could help viruses to maintain a state of latency, although reversible. However, the molecular mechanism underlying such a site-specific integration cannot be excluded and is worthwhile for future investigations. In the scope of DNA virus, it is known that HHV-6A and HHV-6B and the chicken lymphotropic alphaherpesvirus Marek’s disease virus (MDV) can insert specifically into telomere site via the homology-dependent recombination, where the linear double-stranded DNA viruses have variable length of telomere-like repeat regions at either genome end [40,42,43,44]. As shown in Figure 1B, it remains to be determined if the circular *Nimav-1_LVa* genome does harbor one or two tracts of telomeric pentanucleotide (TAACC/GGTTA)_n_.

### 4.2. Endogenized or Free-Living Virus

Although a number of endogenous nimaviruses have been revealed in the genomes of various crustacean species [27,28,80], one compelling question remains, that is whether these endogenous virus sequences are passive relics of some old nimaviruses (“fossilized”), or recent inhabitants in these eukaryotic genomes, from some unidentified free-living viruses, and still possibly possess the capability to proliferate and transmit to different genomes/species under certain circumstances. Currently, at least two cases of endogenous nimaviruses suggest the latter scenario. The first one is the detection of almost identical *Nimav-1_LVa* sequences in the genomes of three shrimp species: *P. vannamei* Kehai No. 1, *P. monodon* isolate Shenzhen (NIUS000000000), and *M. japonicus* isolate Guangxi (NIUR010000000), in spite of the fact that much less *Nimav-1_LVa* is in the latter two shrimp genomes. The second line of evidence is the identification of almost identical (99%) Mj-type nimavirus sequence in *M. japonicus* and *M. latisulcatus* [28]. In light of the unexpected large diversity of virome observed in a single species of marine invertebrate (*P. monodon*) from different geographic locations [81], these data suggest the two nimaviruses or their closest relatives may exist as free-living viruses in nature, except that they may be not so virulent as WSSV (more discussed below).

It is worth noting that endogenous and free-living states are two equally essential stages/phases in the life cycle of some parasitoid viruses [82], such as the polydnavirus *Campoletis sonorensis* ichnovirus (CsIV) [69,70]. The genomes of these viruses, comprising multiple endogenous DNA segments, are endogenously integrated into the genome of the parasitoid wasp (*Campoletis sonorensis*) [69,82], which is parasitic on a host (usually lepidopteran) larva. The virus particles are only replicated (produced) in specific cell types in the female wasp’s reproductive organs and are injected, together with one or more eggs, into the lepidopteran host. In such a system, viral genes are essentially inhibitors of the wasp’s host’s immune system, preventing it from killing the wasp’s injected egg and the immature wasp, until the ultimate death of the parasitized host. This mutualism association or coevolution of virus and parasitoid insect was dated over at least 64 million years [83].

### 4.3. Nimav-1_LVa Encoded Proteins

A total of 117 protein genes, including 97 homology-based and 20 hypothetical genes, have been predicted in the *Nimav-1_LVa* genome if the criterion is set to 70 amino acids long. This number of genes is presumably very close to the actual gene number in *Nimav-1_LVa* because only 16% of the virus genome is intergenic region when long microsatellite regions are excluded. These 117 genes can be generally divided into three sections according to their evolutionary status: (1) 44 nimavirus ancestral/core genes, which are shared in *Nimav-1_LVa* and WSSV, (2) eight genes whose homologs are found in non-WSSV and non-Mj-group nimaviruses, (3) 65 genes whose homologs seemingly only exist in the Mj-group nimaviruses or in the eukaryotic host genome. This division is just for the purpose of expedience because some genuine homologs are inevitably overlooked due to the vast sequence divergence, especially for those smaller genes. Notably, it is possible that in the intergenic region, still exist some smaller protein genes or viral miRNA genes [84,85].

Compared with WSSV, one prominent feature of *Nimav-1_LVa* is that it encodes more than a dozen genes involved in the critical processes in pathogen-host interactions, such as immune responses and/or apoptosis inhibition [86]. These genes/families include *g103* (Hsp70), *g118* (DnaJ, also called Hsp40), *g132* (ubiquitin), *g046* (CHH), *g155* (innexin), *g158* (BAX inhibitor 1 like), *g227* (semaphorin 1A like), *g171* (anti-apoptotic protein), and the six IAPs from the PF2 gene family. We hypothesized that four genes, *g046* (CHH), *g155* (innexin), *g158* (BAX inhibitor 1 like), and *g227* (semaphorin 1A like), were likely derived from cellular genes, but had been harnessed by *Nimav-1_LVa* for its own advantage. This notion was based on the following observations. First, intronic genes are normally very rare in viruses, and all WSSV genes are non-splicing; however, the exon-intron structure is found in *g046* (CHH), *g155* (innexin), and *g158* (BAX inhibitor 1 like) in *Nimav-1_LVa*. Second, to our knowledge, CHH (*g046*) gene has never been reported in a virus genome before. Despite being reported in a few parasitoid viruses, innexin/vinnexin (*g155*) genes are still considered acquired host genes [69,70]. The occurrence of innexin/vinnexin in both *Nimav-1_LVa* and polydnavirus *Campoletis sonorensis* ichnovirus (CsIV) is likely the result of convergent evolution, suggesting *Nimav-1_LVa* virus, to some extent, may not be a virulent virus. Third, all the genes had been reported, or suggested, being involved in immune regulation after virus infection. Lastly, *g155* (innexin), *g158* (BAX inhibitor 1 like), and *g227* (semaphorin 1A like) are all membrane protein genes. In summary, to get a comprehensive perspective on the evolution in *Nimaviridae*, our preliminary results highlight the need for completed assemblies in more endogenous nimaviruses.

## 5. Conclusions

A ~279 Kb contiguous consensus sequence, designated as *Nimav-1_LVa*, was successfully reconstructed from the genome sequence of the whiteleg shrimp *Penaeus vannamei* breed Kehai No. 1. The consensus putatively represented the complete genome of a nimavirus that had been endogenized in the shrimp genome. Out of 117 protein genes, *Nimav-1_LVa* encoded a dozen of genes involved in the host-pathogen interactions, albeit some were acquired host genes. The data suggested *Nimav-1_LVa* virus might take a different strategy than WSSV, aiming at a long-term or benign relationship with the host. The genome of *Nimav-1_LVa* could facilitate a better understanding of evolution in virus family *Nimaviridae* and could also be applicable in the shrimp breeding, traceability of farmed shrimp, WSSV diagnosis, and treatment of WSD [26,87].

## Figures and Tables

**Table 1:** Seven representative nimaviruses of the seven major phylogenetic groups.

Nimaviruses	GenBank Accessions	Size (Kb) ^1^
White spot syndrome virus (WSSV)	AF332093.3	305.119
*C. opilio bacilliform virus* (CoBV)	BDLS01000001 and BDLS01000002	237.1
*M. japonicus* endogenous nimavirus (Mj)	BFCD01000001 and AP010878	~220
*P. monodon* endogenous nimavirus (Pm)	BFCF01000001 to BFCF01000003	191.8
*H. takanoi* endogenous nimavirus (Ht)	BFCC01000001 to BFCC01000006	218.1
*M. ensis* endogenous nimavirus (Me)	BFCE01000001 to BFCE01000010	232.4
*S. intermedium* endogenous nimavirus (Si)	BFCG01000001 to BFCG01000014	189

^1^ Except for the complete genomes of various WSSV strains, the genomes of the other nimaviruses are all incomplete so far. According to Kawato et al. [28], the *M. japonicus* endogenous nimavirus regions in the bacterial artificial chromosome (BAC) clone sequences (AP010878 and BFCD01000001) are added to be only ~220 Kb, excluding the terminal non-viral regions.

**Table 2:** Three types of nimavirus sequences were detected in two shrimp species.

Nimavirus Type	Length (Identity ^1^)	
	*P. monodon*	*M. japonicus*
*Nimav-1_LVa*	>141 Kb (>99%)	>33 Kb (>99%)
Mj-like	>200 Kb (>91%)	>49 Kb (>88%)
Pm-like	>226 Kb (>88%)	>199 Kb (>88%)

^1^ The identity in the parenthesis indicates the minimum sequence identity to the known nimavirus for most majority of the homologous sequences detected in each search.

**Table 3:** A total of 117 protein-coding genes predicted in *Nimav-1_LVa* endogenous nimavirus.

Genes ^1^	CDS start	CDS end	Direction	Protein (AA)	Viral Homolog ^2^	Comment ^3^
**g002**	1388	1990	d	201	g009	PF1
g003	7002	8099	r	217	AKS10635.1	PF2, 4 exons, 2 BIR domains (cd00022)
**g004**	8792	9187	d	132	BFCD01000001.1 (98,829–99,209)	
**g006**	10,037	11,149	d	371	g008	PF1
**g008**	11,219	12,316	d	366	g006	PF1
**g009**	12,527	13,123	r	199	g002	PF1
**g010**	13,467	14,258	r	264	g161	PF1
**g011**	14,749	15,966	d	406	g006	PF1
g012	16,319	19,114	r	725	AKS10635.1	PF2, 4 exons, 3 BIR domains (cd00022), 1 RING-HC_BIRC2_3_7 (cd16713)
g017	19,256	21,711	r	710	AKS10635.1	PF2, 4 exons, 3 BIR domains (cd00022), 1 RING-HC_BIRC2_3_7 (cd16713)
**g021**	24,393	24,710	r	106		
**g022**	27,849	31,305	d	782	AP010878.1 (14,919–16,765)	5 exons
**g026**	31,556	33,238	r	561	AP010878.1 (4458-6026)	
**g027**	33,243	33,857	r	205	AP010878.1 (3787–4395)	
g030	34,488	35,048	r	187	AKS10635.1	PF2, 1 BIR domain (cd00022)
g031	3,5478	37,592	d	705	GBG35399.1	wsv220-like, capsid protein
**g033**	37,838	38,707	d	290		
g034	38,729	39,364	r	212	GBG35402.1	wsv206-like, PF6, containing macro domain (cd02749), a high-affinity ADP-ribose binding module, as shown in GBG35398.
**g036**	39,656	45,952	d	2099	BFCD01000001.1 (62,195–69,574)	
**g038**	46,087	48,771	d	895	BFCD01000001.1 (57,879–62,171)	
**g040**	48,899	49,639	r	247		
g042	49,557	53,573	r	1339	GBG35397.1	wsv026-like
g045	54,485	57,460	d	1138	GBG35396.1	wsv115-like, envelope protein
**g046**	57,833	58,502	r	123		3 exons, CHH-like, containing crust_neurohorm domain (pfam01147)
g047	58,782	60,584	d	448	AKS10635.1	PF2, 4 exons, 2 BIR domains (cd00022), 1 RING-HC_BIRC4_8 (cd16714)
g049	60,851	62,533	d	412	AKS10635.1	PF2, 5 exons, 2 BIR domains (cd00022), 1 RING-HC_BIRC4_8 (cd16714)
**g050**	62,862	64,124	d	421	g051	PF3
**g051**	64,435	65,805	d	457	g050	PF3
**g052**	66,042	69,944	d	1301	BFCD01000001.1 (167,202–171,290)	PF3
**g056**	70,367	71,194	d	276	g269	PF5
**g058**	75,545	76,801	r	419	BFCD01000001.1 (79,831–81,120)	
**g060**	76,972	77,379	r	136	BFCD01000001.1 (79,176–79,601)	
g061	77,382	78,608	r	409	GBG35401.1	wsv415-like, capsid protein
**g062**	78,779	79,048	d	90	BFCD01000001.1 (77,744–77,460)	
g063	79,405	82,122	r	906	GBG35400.1	wsv216-like, envelope protein
**g065**	82,204	82,896	r	231		
**g066**	83,301	83,948	d	216		
**g068**	84,162	84,830	d	223		
g071	86,831	88,126	r	432	GBG35404.1	wsv161-like
g072	88,344	91,454	r	1037	GBG35405.1	wsv011-like, envelope protein
g077	91,612	94,842	r	1077	GBG35406.1	wsv313-like
g081	95,577	96,821	d	415	GBG35407.1	wsv282-like
g083	97,302	115,505	d	6068	GBG35408.1	wsv360-like, capsid protein
g098	115,730	119,659	d	1310	GBG35428.1	wsv037-like, capsid protein
**g103**	119,938	122,058	r	707	GBG35427.1	molecular chaperone DnaK (HSP70) protein domain (COG0443)
**g106**	122,293	123,042	d	250	BFCD01000001.1 (191,561–192,277)	
g107	123,054	123,677	d	208	GBG35426.1	wsv021-like, envelope protein
g108	123,758	127,078	d	1107	GBG35425.1	wsv139-like
g110	127,163	128,206	d	348	GBG35424.1	wsv137-like
g112	128,419	131,400	d	994	GBG35423.1	wsv192-like
g115	131,798	134,191	d	798	GBG35356.1	SCV_095-like, ATP-dependent DNA ligase I (dnl1) domain (TIGR00574) and Poly (ADP-ribose) polymerase and DNA-Ligase Zn-finger (pfam00645)
**g118**	134,634	135,728	d	365		DnaJ/Hsp40 protein, containing DnaJ-class molecular chaperone with C-terminal Zn finger domain (COG0484)
g123	136,705	137,067	d	121	GBG35422.1	wsv136-like
**g125**	137,840	138,184	r	115		
**g126**	139,067	139,678	d	204	BFCD01000001.1 (181,829–182,488)	
g130	140,199	143,633	r	1145	GBG35421.1	wsv271-like, capsid protein
g131	143,809	145,152	d	448	GBG35420.1	wsv131-like
**g132**	145,203	145,433	r	77	BFCD01000001.1 (176,011–176,340)	ubiquitin-like (Ubl) domain (cd01803) found in ubiquitin
g133	145,548	146,687	r	380	GBG35419.1	wsv325-like, envelope protein
**g134**	146,697	147,203	d	169	BFCD01000001.1 (172,895–173,647)	
g135	147,318	148,061	d	248	GBG35417.1	wsv133-like
g136	147,643	148,566	d	308	GBG35418.1	wsv134-like
**g137**	148,715	148,927	r	71		
g139	149,321	149,815	d	165	GBG35402.1	wsv206-like, PF6, containing macro domain (cd02749), a high-affinity ADP-ribose binding module, as shown in GBG35398.
g140	150,049	151,434	d	462	BFCC01000003.1 (801–2426)	wsv112-like, dUTPase, containing deoxyuridine 5’-triphosphate nucleotidohydrolase (dut) domain (TIGR00576)
**g141**	151,587	152,777	d	397	g143	PF1
**g143**	152,974	154,158	d	395	g141	PF1
**g146**	154,360	155,547	d	396	g006	PF1
**g149**	156,541	156,915	d	125		
**g150**	157,204	158,148	r	315		
**g152**	158,385	158,825	d	147		
**g153**	159,042	160,274	d	411		
**g154**	160,465	161,412	d	316		
**g155**	161,589	163,290	d	428	BFCD01000001.1 (51,019–52,483)	4 exons, innexin domain (pfam00876)
**g158**	163,455	165,615	r	274		5 exons, Bax inhibitor (BI)-1 domain (cd10430).
**g161**	165,152	165,772	d	207	g010	PF1
**g162**	166,492	166,767	d	92		
**g163**	167,017	167,787	d	257		
g166	168,473	172,924	d	1484	BBD20107.1	wsv209-like, envelope protein
**g170**	172,897	173,529	d	211	AP010878.1 (53,502–54,038)	
g171	173,590	174,540	d	317	BBD20108.1	wsv267-like, anti-apoptotic protein
**g172**	174,735	175,523	d	263	AP010878.1 (55,291–56,157)	PF4
**g173**	175,556	176,485	d	310	AP010878.1 (55,291–56,157)	PF4
**g175**	176,584	177,510	r	309		
g176	177,844	178,152	d	103	BBD20109.1	wsv293a-like, envelope protein
g177	178,301	183,058	d	1586	GBG35554.1	wsv289-like, capsid protein
g187	183,225	196,220	r	4332	BBD20111.1	wsv343-like
g206	196,741	197,286	d	182	BFCG01000002.1 (22,541–22,065)	SCV_028-like
g208	197,905	199,944	r	680	BBD20112.1	wsv327-like, envelope protein
g211	200,119	202,590	d	824	BBD20113.1	wsv332-like
g213	203,182	204,525	d	448	BBD20114.1	wsv306-like, tegument protein
g217	204,533	206,176	d	548	AP010878.1 (81,486–83,258)	wsv308-like, capsid protein
**g220**	207,060	207,515	d	152	AP010878.1 (84,507–85,430)	
g222	207,948	210,626	d	893	BBD20115.1	wsv285-like
**g223**	210,916	212,928	d	671	AP010878.1 (89,194–91,152)	
g225	218,360	220,426	d	689	GBG35515.1	wsv226-like
**g227**	225,839	227,851	d	671	AP010878.1 (111,284–114,223)	semaphorin 1A (Sema_1A) domain (cd11237)
**g228**	228,056	229,738	d	561	AP010878.1 (109,235–110,863)	
g231	230,064	232,973	d	970	GBG35403.1	wsv035-like, envelope protein
**g234**	233,341	234,681	r	447		In GenBank, part of 234p is computationally predicted as high mobility group protein DSP1-like (XP_027238145.1), 184 AA.
**g236**	234,925	237,936	d	1004	BFCD01000001.1 (86,470–89,643)	
**g240**	241,130	241,501	d	124		
**g241**	241,716	242,537	d	274		
g242	242,655	244,988	r	778	GBG35414.1	wsv303-like
g246	245,095	249,063	r	1323	GBG35413.1	wsv433-like
g251	249,059	249,457	r	133	GBG35412.1	wsv432-like
g252	249,459	251,285	d	609	GBG35411.1	wsv427-like
**g253**	251,758	254,091	d	778	BFCD01000001.1 (134,943–137,015)	
g254	254,266	255,603	d	446	GBG35410.1	wsv423-like, Protein kinase 1
g255	255,856	257,829	d	658	GBG35409.1	wsv440-like
**g257**	258,137	259,543	r	469	g050	PF3
g259	259,757	267,121	r	2455	GBG35416.1	wsv514-like, DNA polymerase
g262	267,305	272,629	d	1775	GBG35415.1	wsv447-like
**g268**	272,942	273,223	d	94		
**g269**	273,601	274,653	d	351	g271	PF5
**g271**	275,034	277,334	d	767	g269	PF5
**g276**	278,291	279,160	d	290	AP010878.1 (55,291–56,157)	PF4

^1^ 65 Mj-group-specific genes are indicated by bold font. ^2^ Viral homologs in this table refer to those present in *Nimav-1_LVa*, WSSV, *Chionoecetes opilio bacilliform virus*, and other endogenous nimaviruses (see Table 1 and methods section). Only the top homologous proteins or coding sequences are listed in this table. The parenthesized coordinates after the accession numbers indicate the homologous coding regions detected by TblastN. ^3^ Exon numbers here refer only to the coding exon. WSSV gene nomenclature indicated with “wsvNNN” is taken from the annotation in AF332093.3 (WSSV-CN strain). PF: paralog families; BIR: Baculovirus Inhibitor of apoptosis protein Repeat; RING-HC: Really Interesting New Gene finger domain of the C3HC4 type; BIRC: baculoviral inhibitor of apoptosis protein repeat containing protein.

## Supplementary Materials

The following are available online at https://www.mdpi.com/2073-4425/11/1/94/s1, Figure S1: Conserved blocks in the alignment of 140p, wsv112 (AAL33116.1), and other homologs, Figure S2: Conserved blocks in the alignment of 225p, wsv226 (AIX03672.1), and other homologs, Figure S3: Conserved blocks in the alignment of 034p, 139p, wsv206 (AAL33210.1), and other homologs, File S1: *Nimav-1_LVa* consensus sequence and 117 encoded protein sequences, Table S1: Shrimp (Kehai No. 1) genomic fragments derived from *Nimav-1_LVa*.
