# Supplementary material for: The Complete Genome of an Endogenous Nimavirus (Nimav-1_LVa) From the Pacific Whiteleg Shrimp Penaeus (Litopenaeus) Vannamei
Source: Genes (Basel). 2020 Jan 14;11(1):94. doi: 10.3390/genes11010094 (PMC7016691; doi:10.3390/genes11010094)
Supplement: Supplementary file 1 [file genes-11-00094-s001.zip › Supplementary Figure legends.pdf]

Supplementary files

Figure legend

FIG S1. Conserved blocks in the alignment of 140p, wsv112 (AAL33116.1) and others homologs. 140p is the protein encoded by gene *g140* in *Nimav-1_LVa*, 140p_Mj denote the homolog of 140p encoded in Mj nimavirus. 140p_Ht denote the homolog of 140p encoded in Ht nimavirus. The other protein and the hosting genome are GAV93148.1 (Chionoecetes opilio bacilliform virus). Numbers after the slash indicate the length of that protein. The locations of the amino acids at the either side of the blocks are indicated by numbers.

FIG S2. Conserved blocks in the alignment of 225p, wsv226 (ALN66200.1) and others homologs. 225p is the protein encoded by gene *g225* in *Nimav-1_LVa*, 225p_Mj denote the homolog of 225p encoded in Mj nimavirus. The other proteins and the hosting genome are GBG35515.1 (Pm nimavirus), GBG35474.1 (Me nimavirus), AKS10608.1 (Md nimavirus), GBG35376.1 (Ht nimavirus), GBG35591.1 (Si nimavirus) and SCV_077+078 (Chionoecetes opilio bacilliform virus), indicating that it is a combined protein sequence from SCV_077 (GAV93197.1) and SCV_078 (GAV93198.1), which are mistaken into two. Numbers after the slash indicate the length of that protein. The locations of the amino acids at the either side of the blocks are indicated by numbers.

FIG S3. Conserved blocks in the alignment of 034p, 139p, wsv206 (AAL33210.1) and others homologs. 034p and 139p are the proteins encoded by gene *g034* and *g139* in *Nimav-1_LVa*, GBG35402.1 and GBG35398.1 are two paralogs of wsv206 in in Mj nimavairus. Numbers after the slash indicate the length of that protein. The locations of the amino acids at the either side of the blocks are indicated by numbers.
