# Supplementary material for: The Complete Genome of an Endogenous Nimavirus (Nimav-1_LVa) From the Pacific Whiteleg Shrimp Penaeus (Litopenaeus) Vannamei
Source: Genes (Basel). 2020 Jan 14;11(1):94. doi: 10.3390/genes11010094 (PMC7016691; doi:10.3390/genes11010094)
Supplement: Supplementary file 1 [file genes-11-00094-s001.zip › Supplementary FigureS1_aln140.pdf]

Fig S1

```
140p/462        YYMGFYKVHND-AFIPKIGLEFSAGSDLYSL-FPCTI  46
140p_Ht/542     YCLGFRKTHTD-AIVPKVATGFSAGSDLHAL-FSCAI  84
140p_Mj/146     SLLKFKKLTEH-AFTPSKGSKFAAGFDLCSA-YDLTI  40
GAV93148.1/322  DILNFQKVHQD-AIIPYRATSGSAGYDVYSRNEEITL  65
AAL33116.1/461  VFMRFAPPGEETALPPRRATPGSVAYDLFPS-EEMDI  43

47 PSNHWAEIRTGVAL-KF        108 ------DASFFLKIRSRSGMALNHNI
85 HAGDQVEVRTGAAI-EF        169 DVIQIKNERFFLKIRSRSGMAANHNI
41 PANGKSLVKTDIQV-EL         57 ------PEGCYGRIAPRSGLSWKYHL
66 CANGQIKIYTGIRVVKF         83 ------PEGCYGRLACSSGWAFANKL
44 EPMGLAKISTGYGIDKF         61 ------PDGCYGQIVSRSGMTWKNNT

NVQAGVIDADYRGEVKVLINNRSS 275
TVQAGVIDADYRGEVKVVLRNDSK 351
NIGAGVVDRDYRGNVGVILFNHAK 100
YIPSGVIDPDYEGEIIVPLCTTSN 126
SVPTGTIDVDYRGELKVILRNHSA 104

276 GNTLRVRAKQPVAMGVVYNIACPI--REII-EIKHPENVKPIDGNM 318
352 DQIFEIEPGMPLAMGVLYDVACPV--TRLIGAIGAIEDHIRIDDGR 395
101 TD-YKVKKGDRIAQLICEKIIYPD--------------------- 123
127 TD-LLIEPNRRVAQLVIERISTPP-----VYETLANGEMVR----N 162
105 EKSVPIRKGTSIAQLIFLRY-CDVEEEQIVYINETTGERTIIDSSS 149
```
