# Supplementary material for: The Complete Genome of an Endogenous Nimavirus (Nimav-1_LVa) From the Pacific Whiteleg Shrimp Penaeus (Litopenaeus) Vannamei
Source: Genes (Basel). 2020 Jan 14;11(1):94. doi: 10.3390/genes11010094 (PMC7016691; doi:10.3390/genes11010094)
Supplement: Supplementary file 1 [file genes-11-00094-s001.zip › Supplementary FigureS2_aln225.pdf]

Fig S2

```
225p/689          EDCKC-KERTSSPFVMDQDT-----EFWNRNTMLRLNIFK  221
225p_Mj/436       PNCKCDRQLRNSVFVSNIDV-----NYWSDDSK-RYNIFR   58
GBG35515.1/1080   QECLSLAEEMGSPF-----NLNGLDTFWVQDKR--KTYLQ  354
GBG35474.1/1113   WDTETASNKFGHPYLPDEESVAIVEKFWNKDNR-KIKYLA  173
AKS10608.1/996    WLFEIAADEFGAPYIPDQKSVAAVENFWAKDNR-KKKYLS  130
GBG35591.1/1081   SLFRVAADEFGAPYLPDQKSINSVENFWGVDDR-KKKYLS  194
SCV_077+078/988   ADHET-AREFGHPYLPDIEKLETVENFWCQE-R-KQRYLG  122
AIX03672.1/936    GICEEAAAN-GRPYLPTLEMRNEVDHFWSQDNR-KLKLLG   72

YF-NNLVLAEFACGKLELFETVDRLRSMLREILTPLLYKI  260    IWDTSYP  294
YFTKNTVFDSFIK-DGDLVNTVEKLRSMLRELLVPFFYQM   97    EWDTAYP  133
HLDNNVVIKSFINGDICAVQFIGLLRSMATEILRPTMYRL  394    RPHSLHS  444
HLVGNYSIKCFIEGTIDTKTCVGFLRSMATAHGCGTIFKL  213    RPESLHP  265
HLRGNHFIECFINGSIDARTCVGMLRSIATEYGYGSVHNV  170    KPDSLHP  221
HLRGNSFIGYFIDGIIDAKTCVGMLRSIATEYGYRSIYNV  234    KPDSLHP  287
HLKGNSVIADFINGDIDVIVFVGLLRSYATEFGYTTLKNL  162    RPNSLHP  213
HFCGNLYVEAFIAGSIDAETCVGFLRSQATGLGYPLLKKL  112    RPSSIQN  153

336 LKCVIALLITPS-NISEVYDLSP    466 KLAIILCGIYGNLKIIAGNSSKLESYFLN
171 IKSIIALLITPS-NFEQVYRPSP    295 KYFIALCGKYENLKFIPGNCSKIENFFLY
486 LQSLICAIVIPC-IVDEVLDLGP    684 RAFISIVGSYGRLHLVTGRINRVYTAFMD
307 LTNLICALIMPAYTLKEMLSLGG    495 RAILTISGSYGWFSLVVGYTSTSDTLFAE
264 LNNIICAIIMPAYTFDEMMALNG    442 RNVLILSGNYGWFGLTLGHILSPDIFFVE
330 LTNLICAIIMPAYTFDEVMALSG    497 RNVLILSGNYGWFGLTLGHTPSPDILFAE
255 ITNLLYAILIPAYSILELLDLGF    463 RSILPLSGSYGWFGLVTGNVQGPDVLFSE
198 LQNLIVAILIPAYTETFVLDTGN    335 RGVLTVSGDYGWFSVIVGSTIMPSVLFYG

KNAFFTKGLSSKSYIHNSREA-IIGI--VFMHNFACLFNDTLHHFKFNDD  568
DNAFFTSGVSCQSYIGNSREH-IIGV--VHMNDFVQLFNDTLNIFKMYKK  398
DNVFFTREDNANIFHCKGLCR-VFGL--VPESRHMDLFRNSRHLFQRMIN  817
SNLFYTQLGYPCWYEADSLSG-VFGN--VELSKHVEVFDGSIQNFYHFLD  646
ENPFVTRMGYPQWYNSESLSA-IFGE--VDLTDHVAALDHTMDKFVKLVE  600
KNPFVTRMGYPQWYNSKGLGD-IFEK--VDLKDHVAALDYTMDKFVKLME  659
HNPFFTRSGTPSWYNPDGLEQ-VFGR--VEYKDHVKVYDDTIHLFKHLAE  626
KNPFFIRHTEPKWYNKNAMCGEVLENVGVTLEQHVRVSDEYMDRFGSLLL  489

EEKLRDNASYLLRLILKNTGRVLYSTGYVWG  604
EQRKRKSCSELLKLILKNSARVLTQRGYVWS  432
MADMRRQAASLIAHLHKISIRQHIRTGRIRG  911
EAEIRLTAQDLYRYMCRMSARLYFSCMLLRG  812
EGECRIAAHDLYKYIYRNSARQYIRSSLIRG  767
DGECRIAAHDLYKYMYRNSARQYIRSSLIRG  826
EGECRVVSHDLLRHLYKSSAKNLIRHTIMRG  785
EAEIRECANELVRELYRASARSYVHDLVLKR  667
```
