# Supplementary material for: The Complete Genome of an Endogenous Nimavirus (Nimav-1_LVa) From the Pacific Whiteleg Shrimp Penaeus (Litopenaeus) Vannamei
Source: Genes (Basel). 2020 Jan 14;11(1):94. doi: 10.3390/genes11010094 (PMC7016691; doi:10.3390/genes11010094)
Supplement: Supplementary file 1 [file genes-11-00094-s001.zip › Supplementary FigureS3_aln34.pdf]

FigS3

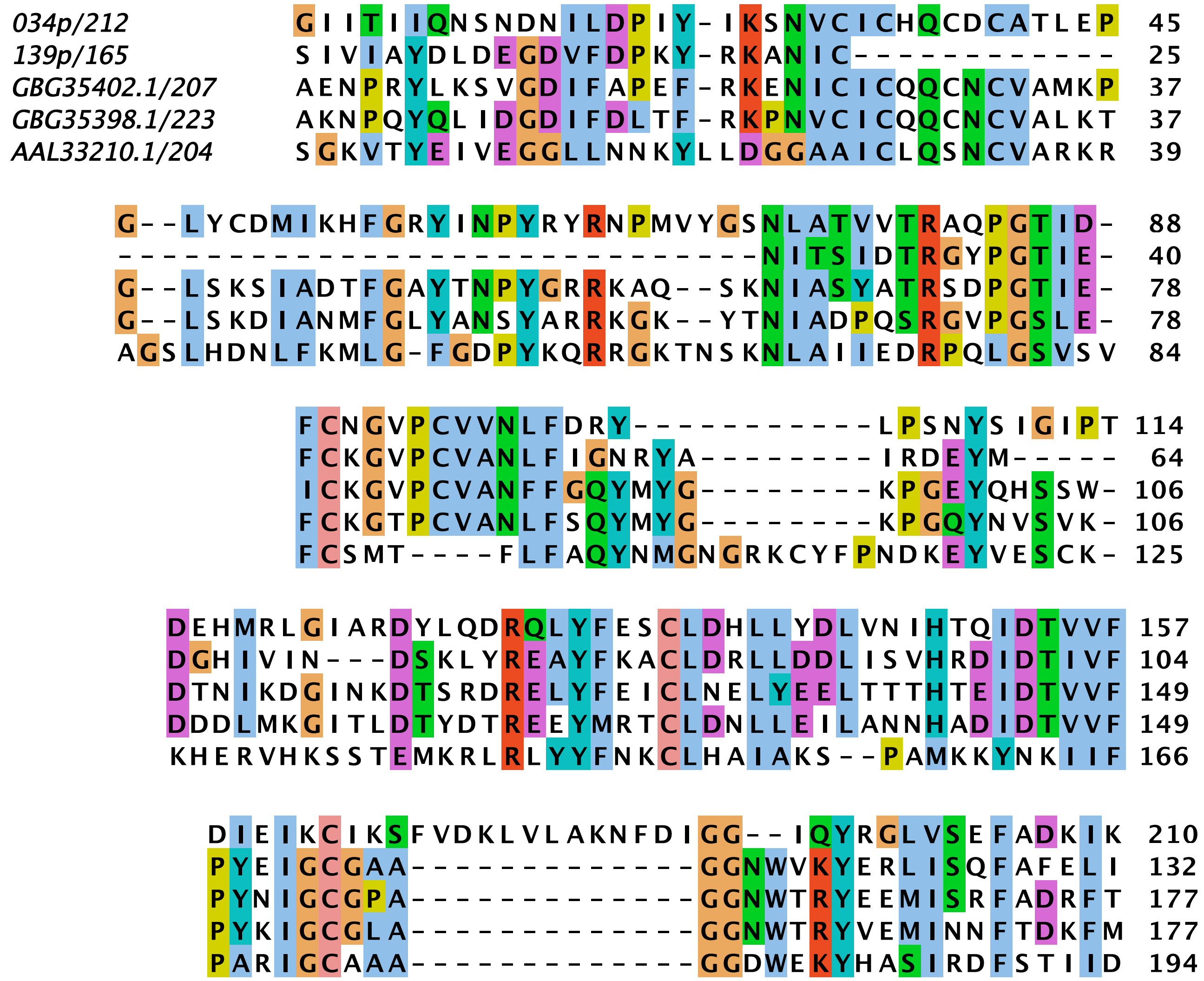
034p/212 GIITIIQNSNDNILDPIY-IKSNVCICHQCDCATLEP 45
139p/165 SIVIAYDLDEGDVFDPKY-RKANIC------------ 25
GBG35402.1/207 AENPRYLKSVGDIFAPEF-RKENICICQQCNCVAMKP 37
GBG35398.1/223 AKNPQYQLIDGDIFDLTF-RKPNVCICQQCNCVALKT 37
AAL33210.1/204 SGKVTYEIVEGGLLNNKYLLDGGAAICLQSNCVARKR 39
G--LYCDMIKHFGRYINPYRYRNPMVYGSNLATVVTRAQPGTID- 88
-----------------------------NITSIDTRGYPGTIE- 40
G--LSKSIADTFGAYTNPYGRRKAQ--SKNIASYATRSDPGTIE- 78
G--LSKDIANMFGLYANSYARRKGK--YTNIADPQSRGVPGSLE- 78
AGSLHDNLFKMLG-FGDPYKQRRGKTNSKNLAIIEDRPQLGSVSV 84
FCNGVPCVVNLFDRY------------LPSNYSIGIPT 114
FCKGVPCVANLFIGNRYA---------IRDEYM----- 64
ICKGVPCVANFFGQYMYG---------KPGEYQHSSW- 106
FCKGTPCVANLFSQYMYG---------KPGQYNVSVK- 106
FCSMT-----FLFAQYNMGNGRKCYFPNDKEYVESCK- 125
DEHMRLGIARDYLQDRQLYFESCLDHLLYDLVNIHTQIDTVVF 157
DGHIVIN---DSKLYREAYFKACLDRLLDDLISVHRDIDTIVF 104
DTNIKDGINKDTSRDRELYFEICLNELYEELTTTHTEIDTVVF 149
DDDLMKGITLDTYDTREEYMRTCLDNLLEILANNHADIDTVVF 149
KHERVHKSSTEMKRLRLYYFNKCLHAIAKS--PAMKKYNKIIF 166
DIEIKCIKSFVDKLVLAKNFDIGG--IQYRGLVSEFADKIK 210
PYEIGCGAA-------------GGNWVKYERLISQFAFELI 132
PYNIGCGPA-------------GGNWTRYEEMISRFADRFT 177
PYKIGCGLA-------------GGNWTRYVEMINNFTDKFM 177
PARIGCAAA-------------GGDWEKYHASIRDFSTIID 194
