# Supplementary material for: The Complete Genome of an Endogenous Nimavirus (Nimav-1_LVa) From the Pacific Whiteleg Shrimp Penaeus (Litopenaeus) Vannamei
Source: Genes (Basel). 2020 Jan 14;11(1):94. doi: 10.3390/genes11010094 (PMC7016691; doi:10.3390/genes11010094)
Supplement: Supplementary file 1 [file genes-11-00094-s001.zip › Supplementary TableS1-fragment.pdf]

Talbe S1: Genomic fragments derived from Nimav-1_LVa in order of descending length. Fragment with bold name are those illustrated in figure 1.

Fragment	Loci	L-star	L-end	virus	v-star	v-end	strand	identity	Length
**F259**	NW_020871249.1	409780	456533	Nimav-1_LVa	114474	161156	d	0.9949	46683
**F268**	NW_020871279.1	70471	112901	Nimav-1_LVa	161402	203789	d	0.9969	42388
**F375**	NW_020872607.1	395519	433268	Nimav-1_LVa	118207	156129	d	0.9959	37923
**F363**	NW_020872505.1	203037	239990	Nimav-1_LVa	192177	229348	d	0.9951	37172
**F194**	NW_020870348.1	297574	333166	Nimav-1_LVa	1	35601	d	0.9954	35601
**F276**	NW_020871384.1	28926	63318	Nimav-1_LVa	189899	224374	d	0.9897	34476
**F120**	NW_020870086.1	109003	143107	Nimav-1_LVa	144190	178448	d	0.9969	34259
**F341**	NW_020872253.1	829034	862550	Nimav-1_LVa	56108	89833	d	0.9713	33726
**F138**	NW_020870086.1	331695	364870	Nimav-1_LVa	244280	277761	d	0.9936	33482
**F192**	NW_020870319.1	537827	570208	Nimav-1_LVa	236503	268884	c	0.9973	32382
**F349**	NW_020872253.1	958712	989885	Nimav-1_LVa	94358	125802	d	0.9932	31445
**F359**	NW_020872350.1	91571	122397	Nimav-1_LVa	114645	145594	c	0.9955	30950

**F141**	NW_020870185.1	412648	441464	Nimav-1_LVa	135801	164891	c	0.9778	29091
**F020**	NW_020868486.1	380179	407460	Nimav-1_LVa	234722	262488	c	0.9933	27767
**F202**	NW_020870398.1	142969	170294	Nimav-1_LVa	9961	37650	d	0.9921	27690
**F340**	NW_020872253.1	801712	828925	Nimav-1_LVa	73941	101447	d	0.9908	27507
**F329**	NW_020872253.1	625915	652962	Nimav-1_LVa	71542	98323	d	0.9817	26782
**F269**	NW_020871279.1	112916	139632	Nimav-1_LVa	205226	231984	d	0.9946	26759
F182	NW_020870259.1	252256	277847	Nimav-1_LVa	247660	273517	c	0.9963	25858
**F170**	NW_020870185.1	591020	615779	Nimav-1_LVa	24352	49341	d	0.9929	24990
**F052**	NW_020868730.1	92929	117620	Nimav-1_LVa	44635	69399	c	0.9957	24765
F348	NW_020872253.1	934847	958580	Nimav-1_LVa	70536	94037	d	0.9892	23502
F289	NW_020871640.1	130841	153404	Nimav-1_LVa	110172	132928	d	0.9815	22757
F060	NW_020869103.1	420666	443332	Nimav-1_LVa	141895	164621	c	0.9974	22727
F330	NW_020872253.1	673600	696257	Nimav-1_LVa	78984	101723	d	0.9947	22740
**F339**	NW_020872253.1	779215	801611	Nimav-1_LVa	64250	86765	d	0.9931	22516

F116	NW_020870086.1	69928	92341	Nimav-1_LVa	89616	112087	d	0.9959	22472
F242	NW_020871127.1	394166	416176	Nimav-1_LVa	244510	266782	d	0.9905	22273
**F353**	NW_020872253.1	1020118	1041815	Nimav-1_LVa	94064	116066	c	0.9951	22003
F327	NW_020872253.1	582933	604722	Nimav-1_LVa	72449	94393	d	0.9903	21945
F223	NW_020870747.1	308573	330115	Nimav-1_LVa	73906	95273	d	0.9898	21368
F016	NW_020868486.1	338874	359880	Nimav-1_LVa	242203	263481	d	0.9933	21279
**F038**	NW_020868486.1	647228	667915	Nimav-1_LVa	45540	66420	c	0.9879	20881
**F064**	NW_020869331.1	131429	152178	Nimav-1_LVa	259084	279905	d	0.9968	20822
F125	NW_020870086.1	168464	188942	Nimav-1_LVa	150266	170807	c	0.9938	20542
F335	NW_020872253.1	732099	751604	Nimav-1_LVa	76178	95776	d	0.9948	19599
F303	NW_020871803.1	29035	48337	Nimav-1_LVa	59340	78876	c	0.9904	19537
F025	NW_020868486.1	469316	488454	Nimav-1_LVa	143024	162150	c	0.9864	19127
F091	NW_020869804.1	265397	284232	Nimav-1_LVa	127098	146085	c	0.9940	18988
F333	NW_020872253.1	708364	727157	Nimav-1_LVa	82059	100990	d	0.9946	18932

**F364**	NW_020872505.1	240095	258708	Nimav-1_LVa	217341	236122	d	0.9902	18782
F187	NW_020870265.1	174162	192501	Nimav-1_LVa	15757	34161	c	0.9901	18405
F270	NW_020871279.1	139905	158418	Nimav-1_LVa	232452	250840	d	0.9935	18389
F144	NW_020870185.1	454291	472511	Nimav-1_LVa	107353	125664	c	0.9895	18312
F204	NW_020870398.1	178615	196627	Nimav-1_LVa	44638	62878	d	0.9848	18241
F044	NW_020868486.1	736532	754124	Nimav-1_LVa	235880	253955	c	0.9846	18076
**F127**	NW_020870086.1	210485	228262	Nimav-1_LVa	156242	174265	d	0.9924	18024
F321	NW_020872253.1	480882	498595	Nimav-1_LVa	44728	62727	d	0.9850	18000
**F037**	NW_020868486.1	544073	561355	Nimav-1_LVa	1	17531	c	0.9927	17531
**F266**	NW_020871249.1	491419	508982	Nimav-1_LVa	185979	203499	d	0.9904	17521
F250	NW_020871249.1	215934	233058	Nimav-1_LVa	246486	263888	c	0.9924	17403
F326	NW_020872253.1	565462	582745	Nimav-1_LVa	83028	100427	d	0.9950	17400
F233	NW_020871127.1	341442	358438	Nimav-1_LVa	262608	279905	c	0.9607	17298
**F315**	NW_020872166.1	127642	144638	Nimav-1_LVa	262608	279905	c	0.9894	17298

F053	NW_020868730.1	117922	134701	Nimav-1_LVa	25881	43027	c	0.9872	17147
F011	NW_020868486.1	281684	298608	Nimav-1_LVa	191079	208123	d	0.9923	17045
F157	NW_020870185.1	535337	552566	Nimav-1_LVa	2807	19770	c	0.9865	16964
F024	NW_020868486.1	447658	464418	Nimav-1_LVa	150598	167432	c	0.9943	16835
F322	NW_020872253.1	510564	526995	Nimav-1_LVa	57708	74380	d	0.9885	16673
F305	NW_020871803.1	82567	99115	Nimav-1_LVa	8165	24785	c	0.9815	16621
F336	NW_020872253.1	751705	768008	Nimav-1_LVa	79055	95483	d	0.9947	16429
F346	NW_020872253.1	908948	924995	Nimav-1_LVa	21423	37650	d	0.9925	16228
F281	NW_020871640.1	51524	67291	Nimav-1_LVa	49702	65800	d	0.9931	16099
F205	NW_020870398.1	208127	223965	Nimav-1_LVa	35967	51781	c	0.9668	15815
**F374**	NW_020872515.1	1081798	1097442	Nimav-1_LVa	1	15605	d	0.9950	15605
F190	NW_020870265.1	198003	213484	Nimav-1_LVa	1	15586	c	0.9946	15586
F156	NW_020870185.1	520205	535335	Nimav-1_LVa	5712	20945	c	0.9764	15234
F225	NW_020870747.1	333884	348901	Nimav-1_LVa	103513	118628	d	0.9948	15116

F227	NW_020870747.1	351320	366321	Nimav-1_LVa	119553	134648	d	0.9934	15096
F302	NW_020871791.1	165755	180395	Nimav-1_LVa	262348	277175	d	0.9931	14828
F015	NW_020868486.1	324400	338873	Nimav-1_LVa	235931	250712	d	0.9906	14782
F296	NW_020871791.1	118843	133544	Nimav-1_LVa	246449	261234	d	0.9941	14786
F256	NW_020871249.1	285536	300085	Nimav-1_LVa	262481	277177	d	0.9942	14697
F106	NW_020869804.1	390929	405555	Nimav-1_LVa	151400	166046	d	0.9938	14647
F352	NW_020872253.1	1005314	1019863	Nimav-1_LVa	102694	117199	c	0.9811	14506
F244	NW_020871127.1	418207	432598	Nimav-1_LVa	265426	279904	d	0.9940	14479
F245	NW_020871127.1	433149	447467	Nimav-1_LVa	265445	279905	d	0.9957	14461
F251	NW_020871249.1	233059	247455	Nimav-1_LVa	108078	122542	c	0.9963	14465
F275	NW_020871295.1	649277	663506	Nimav-1_LVa	5154	19459	c	0.9939	14306
F328	NW_020872253.1	604723	618853	Nimav-1_LVa	79925	94239	c	0.9801	14315
F304	NW_020871803.1	48427	62486	Nimav-1_LVa	45304	59535	c	0.9800	14232
F101	NW_020869804.1	339915	353972	Nimav-1_LVa	94897	109052	d	0.9941	14156

F345	NW_020872253.1	894978	908832	Nimav-1_LVa	71542	85477	d	0.9781	13936
F271	NW_020871279.1	173999	187865	Nimav-1_LVa	251348	265126	d	0.9774	13779
F030	NW_020868486.1	509481	522833	Nimav-1_LVa	95571	109052	c	0.9952	13482
F126	NW_020870086.1	188944	202239	Nimav-1_LVa	144261	157690	c	0.9916	13430
F183	NW_020870265.1	135963	148913	Nimav-1_LVa	48695	61972	c	0.9916	13278
F093	NW_020869804.1	297122	310161	Nimav-1_LVa	108375	121482	c	0.9969	13108
F128	NW_020870086.1	236258	249219	Nimav-1_LVa	187028	200073	d	0.9928	13046
F323	NW_020872253.1	533177	546001	Nimav-1_LVa	76961	89862	d	0.9868	12902
F078	NW_020869739.1	60314	73167	Nimav-1_LVa	56358	69228	c	0.9953	12871
F149	NW_020870185.1	486475	499298	Nimav-1_LVa	89365	102221	c	0.9965	12857
F255	NW_020871249.1	273042	285532	Nimav-1_LVa	262772	275507	d	0.9892	12736
F058	NW_020868841.1	181360	193855	Nimav-1_LVa	123191	135889	c	0.9875	12699
F293	NW_020871640.1	181213	193816	Nimav-1_LVa	127183	139799	c	0.9954	12617
F307	NW_020871956.1	202983	215458	Nimav-1_LVa	45164	57770	c	0.9817	12607

F042	NW_020868486.1	696924	709566	Nimav-1_LVa	1246	13825	c	0.9879	12580
F260	NW_020871249.1	456538	469038	Nimav-1_LVa	89666	102221	d	0.9933	12556
F061	NW_020869103.1	443341	455867	Nimav-1_LVa	141959	154495	d	0.9972	12537
F295	NW_020871791.1	106534	118842	Nimav-1_LVa	241708	254056	d	0.9952	12349
F358	NW_020872350.1	78375	90636	Nimav-1_LVa	146909	159241	c	0.9957	12333
**F186**	NW_020870265.1	162360	174155	Nimav-1_LVa	34413	46586	c	0.9840	12174
F112	NW_020870030.1	241087	253086	Nimav-1_LVa	194865	206977	d	0.9942	12113
F254	NW_020871249.1	260961	273040	Nimav-1_LVa	246486	258556	d	0.9945	12071
F277	NW_020871384.1	91614	103610	Nimav-1_LVa	242632	254689	d	0.9943	12058
F080	NW_020869739.1	74030	85917	Nimav-1_LVa	45543	57570	c	0.9849	12028
F111	NW_020870030.1	229166	241052	Nimav-1_LVa	194907	206933	c	0.9891	12027
F132	NW_020870086.1	274124	286120	Nimav-1_LVa	212385	224388	d	0.9865	12004
F236	NW_020871127.1	364953	376615	Nimav-1_LVa	242206	254220	c	0.9870	12015
F248	NW_020871249.1	202272	213793	Nimav-1_LVa	266442	278441	c	0.9889	12000

F306	NW_020871803.1	99116	110938	Nimav-1_LVa	8720	20732	c	0.9915	12013
F274	NW_020871279.1	203657	215305	Nimav-1_LVa	265107	277019	d	0.9890	11913
F092	NW_020869804.1	284250	295961	Nimav-1_LVa	115055	126878	c	0.9913	11824
F110	NW_020869942.1	96184	107801	Nimav-1_LVa	6677	18394	d	0.9925	11718
F039	NW_020868486.1	667932	679530	Nimav-1_LVa	32815	44487	c	0.9936	11673
F088	NW_020869739.1	103354	114805	Nimav-1_LVa	25999	37650	d	0.9880	11652
F090	NW_020869804.1	253812	265387	Nimav-1_LVa	146282	157866	c	0.9887	11585
F104	NW_020869804.1	371619	383075	Nimav-1_LVa	133758	145201	d	0.9951	11444
F286	NW_020871640.1	107676	119026	Nimav-1_LVa	90157	101541	d	0.9938	11385
F300	NW_020871791.1	153188	164448	Nimav-1_LVa	247576	258892	d	0.9943	11317
F019	NW_020868486.1	368332	379511	Nimav-1_LVa	264761	275931	c	0.9979	11171
F077	NW_020869739.1	47374	58343	Nimav-1_LVa	72607	83728	c	0.9508	11122
F009	NW_020868315.1	349669	360506	Nimav-1_LVa	248398	259492	d	0.9880	11095
F008	NW_020868315.1	338885	349659	Nimav-1_LVa	242208	253204	d	0.9900	10997

F108	NW_020869804.1	425138	436050	Nimav-1_LVa	187565	198538	d	0.9958	10974
F050	NW_020868486.1	817315	828510	Nimav-1_LVa	40005	50942	c	0.9680	10938
F072	NW_020869739.1	30	10583	Nimav-1_LVa	204216	215133	c	0.9858	10918
F191	NW_020870319.1	527024	537822	Nimav-1_LVa	269048	279905	c	0.9950	10858
F012	NW_020868486.1	298639	309291	Nimav-1_LVa	208260	219099	d	0.9864	10840
F273	NW_020871279.1	190278	201008	Nimav-1_LVa	267256	278083	d	0.9916	10828
F049	NW_020868486.1	800802	811474	Nimav-1_LVa	50928	61668	c	0.9933	10741
F174	NW_020870249.1	184864	195581	Nimav-1_LVa	25939	36654	c	0.9893	10716
F283	NW_020871640.1	76254	86896	Nimav-1_LVa	53412	64124	d	0.9940	10713
F299	NW_020871791.1	142758	153187	Nimav-1_LVa	252873	263591	d	0.9655	10719
F252	NW_020871249.1	247470	258090	Nimav-1_LVa	107986	118638	d	0.9958	10653
F351	NW_020872253.1	992761	1003335	Nimav-1_LVa	118335	128962	c	0.9940	10628
F070	NW_020869429.1	254346	264880	Nimav-1_LVa	219478	230033	c	0.9930	10556
F280	NW_020871640.1	37587	47933	Nimav-1_LVa	34980	45452	d	0.9800	10473

F062	NW_020869297.1	113527	123785	Nimav-1_LVa	249810	260193	d	0.9944	10384
F168	NW_020870185.1	573895	584122	Nimav-1_LVa	6581	16951	d	0.9906	10371
F076	NW_020869739.1	31352	41554	Nimav-1_LVa	92395	102614	c	0.9970	10220
F099	NW_020869804.1	327603	337628	Nimav-1_LVa	91733	101849	c	0.9956	10117
F241	NW_020871127.1	384171	394159	Nimav-1_LVa	233878	243906	d	0.9957	10029
F308	NW_020871956.1	225464	235527	Nimav-1_LVa	27097	37071	c	0.9906	9975
F180	NW_020870259.1	236056	245868	Nimav-1_LVa	1	9875	c	0.9933	9875
F343	NW_020872253.1	872469	882317	Nimav-1_LVa	76180	86069	c	0.9849	9890
F342	NW_020872253.1	862666	872413	Nimav-1_LVa	76287	86124	d	0.9943	9838
F347	NW_020872253.1	924996	934732	Nimav-1_LVa	76180	86006	d	0.9938	9827
F140	NW_020870086.1	378001	387742	Nimav-1_LVa	3770	13570	d	0.9807	9801
F103	NW_020869804.1	361916	371599	Nimav-1_LVa	121498	131175	d	0.9973	9678
F344	NW_020872253.1	882318	891891	Nimav-1_LVa	27989	37650	c	0.9950	9662
F043	NW_020868486.1	715262	724761	Nimav-1_LVa	266391	276016	c	0.9909	9626

**F265**	NW_020871249.1	481461	491009	Nimav-1_LVa	174499	184126	d	0.9970	9628
F228	NW_020870984.1	618598	628327	Nimav-1_LVa	31520	41125	d	0.9809	9606
F285	NW_020871640.1	93959	103351	Nimav-1_LVa	71717	81098	d	0.9852	9382
F041	NW_020868486.1	687961	696895	Nimav-1_LVa	14376	23660	c	0.9835	9285
F291	NW_020871640.1	162635	171846	Nimav-1_LVa	142176	151416	d	0.9973	9241
F287	NW_020871640.1	119968	129116	Nimav-1_LVa	102779	111941	d	0.9959	9163
F357	NW_020872350.1	69240	78371	Nimav-1_LVa	150159	159320	c	0.9957	9162
F282	NW_020871640.1	67301	76219	Nimav-1_LVa	45187	54294	d	0.9784	9108
F360	NW_020872350.1	122541	131485	Nimav-1_LVa	106773	115807	c	0.9926	9035
F074	NW_020869739.1	17547	26402	Nimav-1_LVa	112476	121495	c	0.9912	9020
F047	NW_020868486.1	788500	797138	Nimav-1_LVa	65910	74836	c	0.9794	8927
F232	NW_020871091.1	64680	73558	Nimav-1_LVa	270994	279905	d	0.9954	8912
F137	NW_020870086.1	310466	319248	Nimav-1_LVa	233695	242478	c	0.9969	8784
F292	NW_020871640.1	171860	180589	Nimav-1_LVa	142682	151433	c	0.9939	8752

F338	NW_020872253.1	770466	779108	Nimav-1_LVa	76178	84874	d	0.9935	8697
F121	NW_020870086.1	143109	151880	Nimav-1_LVa	169782	178440	c	0.9785	8659
F316	NW_020872166.1	144640	152997	Nimav-1_LVa	252559	261205	c	0.9922	8647
F198	NW_020870348.1	364681	373282	Nimav-1_LVa	31983	40536	d	0.9800	8554
**F069**	NW_020869429.1	245827	254230	Nimav-1_LVa	230346	238811	c	0.9953	8466
F171	NW_020870185.1	615785	624125	Nimav-1_LVa	49638	58001	d	0.9936	8364
F279	NW_020871384.1	116684	124733	Nimav-1_LVa	251510	259861	d	0.9697	8352
F150	NW_020870185.1	501124	509298	Nimav-1_LVa	45853	54152	c	0.9879	8300
F032	NW_020868486.1	523844	532030	Nimav-1_LVa	45862	54143	c	0.9891	8282
F131	NW_020870086.1	266201	274109	Nimav-1_LVa	213502	221722	d	0.9813	8221
F278	NW_020871384.1	104148	112308	Nimav-1_LVa	255741	263974	d	0.9934	8234
F029	NW_020868486.1	501521	509480	Nimav-1_LVa	112483	120692	c	0.9898	8210
F176	NW_020870249.1	197540	205288	Nimav-1_LVa	15110	23229	c	0.9873	8120
F001	NW_020868315.1	300001	307958	Nimav-1_LVa	194243	202264	c	0.9937	8022

F096	NW_020869804.1	316278	324189	Nimav-1_LVa	93523	101517	c	0.9919	7995
F102	NW_020869804.1	353973	361915	Nimav-1_LVa	112483	120385	d	0.9725	7903
F124	NW_020870086.1	160781	168434	Nimav-1_LVa	171172	178862	c	0.9931	7691
F196	NW_020870348.1	347332	355131	Nimav-1_LVa	38835	46496	c	0.9906	7662
F325	NW_020872253.1	557829	565461	Nimav-1_LVa	83275	90924	c	0.9954	7650
F040	NW_020868486.1	679927	687157	Nimav-1_LVa	24540	31964	c	0.9884	7425
F115	NW_020870030.1	265267	272241	Nimav-1_LVa	204216	211307	d	0.9867	7092
F284	NW_020871640.1	87005	93725	Nimav-1_LVa	64387	71451	d	0.9870	7065
F109	NW_020869804.1	436306	443163	Nimav-1_LVa	199338	206335	d	0.9934	6998
F142	NW_020870185.1	445728	452678	Nimav-1_LVa	120475	127397	c	0.9922	6923
F169	NW_020870185.1	584141	590933	Nimav-1_LVa	17228	24046	d	0.9960	6819
F331	NW_020872253.1	697116	703806	Nimav-1_LVa	78595	85295	d	0.9925	6701
F023	NW_020868486.1	439640	446332	Nimav-1_LVa	192192	198822	c	0.9749	6631
F324	NW_020872253.1	551088	557726	Nimav-1_LVa	90993	97625	d	0.9931	6633

F117	NW_020870086.1	92342	98557	Nimav-1_LVa	114246	120692	d	0.9820	6447
F377	NW_020872607.1	433755	439869	Nimav-1_LVa	157134	163531	d	0.9861	6398
F056	NW_020868730.1	140906	147042	Nimav-1_LVa	11326	17714	c	0.9826	6389
F181	NW_020870259.1	245943	252255	Nimav-1_LVa	273518	279905	c	0.9946	6388
F113	NW_020870030.1	253463	259741	Nimav-1_LVa	115043	121336	d	0.9944	6294
F123	NW_020870086.1	154278	160525	Nimav-1_LVa	172704	178949	d	0.9939	6246
F129	NW_020870086.1	249661	255969	Nimav-1_LVa	200630	206862	d	0.9816	6233
F045	NW_020868486.1	773944	779699	Nimav-1_LVa	207880	213949	c	0.9831	6070
F221	NW_020870618.1	426231	432317	Nimav-1_LVa	198643	204722	c	0.9980	6080
F003	NW_020868315.1	313139	318778	Nimav-1_LVa	201060	206901	d	0.9864	5842
F195	NW_020870348.1	341396	347331	Nimav-1_LVa	40512	46372	d	0.9888	5861
F165	NW_020870185.1	563597	569332	Nimav-1_LVa	6677	12506	d	0.9901	5830
F135	NW_020870086.1	303908	309517	Nimav-1_LVa	239059	244799	d	0.9804	5741
F313	NW_020871956.1	246792	252312	Nimav-1_LVa	8283	13827	c	0.9957	5545

F073	NW_020869739.1	10586	16048	Nimav-1_LVa	122604	128118	c	0.9878	5515
F290	NW_020871640.1	153801	159327	Nimav-1_LVa	132984	138493	d	0.9891	5510
F082	NW_020869739.1	88381	93722	Nimav-1_LVa	45853	51321	c	0.9850	5469
F017	NW_020868486.1	361735	366928	Nimav-1_LVa	271785	277125	c	0.9864	5341
F002	NW_020868315.1	307959	313050	Nimav-1_LVa	194352	199681	d	0.9912	5330
F071	NW_020869429.1	264944	270119	Nimav-1_LVa	214032	219177	c	0.9900	5146
F311	NW_020871956.1	238275	243389	Nimav-1_LVa	18080	23229	c	0.9904	5150
F105	NW_020869804.1	383087	388149	Nimav-1_LVa	145505	150577	d	0.9921	5073
F022	NW_020868486.1	433919	438949	Nimav-1_LVa	199403	204454	c	0.9863	5052
F094	NW_020869804.1	310220	315152	Nimav-1_LVa	103533	108526	c	0.9933	4994
F267	NW_020871249.1	511314	516314	Nimav-1_LVa	204517	209505	d	0.9813	4989
F258	NW_020871249.1	404826	409779	Nimav-1_LVa	106976	111941	d	0.9978	4966
F005	NW_020868315.1	331041	335768	Nimav-1_LVa	233899	238800	d	0.9888	4902
F114	NW_020870030.1	260506	265266	Nimav-1_LVa	123168	128052	d	0.9922	4885

F075	NW_020869739.1	26403	31213	Nimav-1_LVa	104166	109045	c	0.9954	4880
F230	NW_020870984.1	664761	669460	Nimav-1_LVa	195	5071	c	0.9850	4877
F334	NW_020872253.1	727268	732098	Nimav-1_LVa	32780	37648	d	0.9952	4869
F319	NW_020872253.1	467246	471978	Nimav-1_LVa	31365	36212	d	0.9793	4848
F231	NW_020870984.1	669984	674806	Nimav-1_LVa	1	4802	d	0.9759	4802
F373	NW_020872515.1	1075499	1080134	Nimav-1_LVa	275260	279905	d	0.9970	4646
F172	NW_020870185.1	624196	628724	Nimav-1_LVa	53411	58017	c	0.9899	4607
F147	NW_020870185.1	478254	482686	Nimav-1_LVa	152672	157212	c	0.9894	4541
F332	NW_020872253.1	703807	708253	Nimav-1_LVa	80567	85095	c	0.9726	4529
F185	NW_020870265.1	155503	159783	Nimav-1_LVa	39469	43791	c	0.9893	4323
F059	NW_020868866.1	442172	446421	Nimav-1_LVa	171224	175476	d	0.9988	4253
F004	NW_020868315.1	326932	331040	Nimav-1_LVa	224612	228832	d	0.9617	4221
F146	NW_020870185.1	474061	478253	Nimav-1_LVa	162639	166813	c	0.9926	4175
F264	NW_020871249.1	477275	481460	Nimav-1_LVa	162630	166809	d	0.9899	4180

F027	NW_020868486.1	494522	498477	Nimav-1_LVa	136592	140693	c	0.9750	4102
F055	NW_020868730.1	136698	140656	Nimav-1_LVa	19191	23229	c	0.9778	4039
F197	NW_020870348.1	355670	359784	Nimav-1_LVa	33995	37981	c	0.9890	3987
F257	NW_020871249.1	400908	404738	Nimav-1_LVa	102707	106655	d	0.9891	3949
F013	NW_020868486.1	310156	313946	Nimav-1_LVa	220468	224388	d	0.9838	3921
F173	NW_020870185.1	628725	632597	Nimav-1_LVa	63268	67145	d	0.9969	3878
F261	NW_020871249.1	469041	472906	Nimav-1_LVa	148664	152560	d	0.9951	3897
F240	NW_020871127.1	380293	384148	Nimav-1_LVa	233575	237442	c	0.9940	3868
F246	NW_020871199.1	64315	68108	Nimav-1_LVa	9961	13829	c	0.9848	3869
F314	NW_020871956.1	252317	256112	Nimav-1_LVa	12308	16109	c	0.9515	3802
F148	NW_020870185.1	482688	486472	Nimav-1_LVa	148664	152453	c	0.9921	3790
F224	NW_020870747.1	330125	333882	Nimav-1_LVa	98394	102173	d	0.9955	3780
F234	NW_020871127.1	358439	362114	Nimav-1_LVa	257468	261214	c	0.9897	3747
F262	NW_020871249.1	472907	476420	Nimav-1_LVa	152653	156217	d	0.9943	3565

F312	NW_020871956.1	243405	246785	Nimav-1_LVa	14348	17860	c	0.9886	3513
F048	NW_020868486.1	797375	800794	Nimav-1_LVa	61844	65340	c	0.9890	3497
F021	NW_020868486.1	407762	411183	Nimav-1_LVa	230341	233808	c	0.9766	3468
F133	NW_020870086.1	291468	294968	Nimav-1_LVa	227962	231380	d	0.9812	3419
F203	NW_020870398.1	170295	173716	Nimav-1_LVa	76180	79594	d	0.9895	3415
F155	NW_020870185.1	517113	520201	Nimav-1_LVa	21170	24525	c	0.9781	3356
F167	NW_020870185.1	570703	573894	Nimav-1_LVa	3025	6307	d	0.9855	3283
F189	NW_020870265.1	194730	198000	Nimav-1_LVa	12388	15674	d	0.9945	3287
F361	NW_020872403.1	739276	742301	Nimav-1_LVa	90431	93472	c	0.9921	3042
F294	NW_020871640.1	204695	207641	Nimav-1_LVa	118002	120970	c	0.9939	2969
F247	NW_020871199.1	70315	73102	Nimav-1_LVa	6497	9429	c	0.9564	2933
F057	NW_020868730.1	147043	149905	Nimav-1_LVa	7301	10185	c	0.9919	2885
F350	NW_020872253.1	989886	992757	Nimav-1_LVa	126167	129043	d	0.9951	2877
F153	NW_020870185.1	512018	514815	Nimav-1_LVa	27572	30418	c	0.9843	2847

F201	NW_020870398.1	138106	140901	Nimav-1_LVa	6581	9429	d	0.9705	2849
F033	NW_020868486.1	532032	534817	Nimav-1_LVa	27568	30393	c	0.9871	2826
F035	NW_020868486.1	537079	539648	Nimav-1_LVa	21665	24499	c	0.9853	2835
F179	NW_020870249.1	216234	219081	Nimav-1_LVa	1	2803	c	0.9621	2803
F253	NW_020871249.1	258185	260960	Nimav-1_LVa	119752	122542	d	0.9892	2791
F160	NW_020870185.1	556296	559229	Nimav-1_LVa	2066	4844	d	0.9568	2779
F199	NW_020870398.1	130899	133383	Nimav-1_LVa	1	2602	d	0.9791	2602
F034	NW_020868486.1	534818	537074	Nimav-1_LVa	24806	27369	c	0.9741	2564
F139	NW_020870086.1	375172	377686	Nimav-1_LVa	1	2538	d	0.9893	2538
F154	NW_020870185.1	514816	517112	Nimav-1_LVa	24816	27369	c	0.9688	2554
F006	NW_020868315.1	335777	338307	Nimav-1_LVa	239289	241781	c	0.9530	2493
F238	NW_020871127.1	377191	379703	Nimav-1_LVa	239289	241781	d	0.9601	2493
F081	NW_020869739.1	85926	88369	Nimav-1_LVa	42050	44509	c	0.9951	2460
F026	NW_020868486.1	491212	493417	Nimav-1_LVa	137752	140173	d	0.9682	2422

F046	NW_020868486.1	779701	782106	Nimav-1_LVa	194698	197132	c	0.9929	2435
F014	NW_020868486.1	313947	316316	Nimav-1_LVa	224627	227006	d	0.9975	2380
F107	NW_020869804.1	408078	410507	Nimav-1_LVa	167697	170082	d	0.9750	2386
F184	NW_020870265.1	148927	151307	Nimav-1_LVa	46679	49065	d	0.9983	2387
F226	NW_020870747.1	348949	351317	Nimav-1_LVa	119836	122208	d	0.9979	2373
F298	NW_020871791.1	134533	136912	Nimav-1_LVa	264123	266498	d	0.9958	2376
F356	NW_020872350.1	62165	64483	Nimav-1_LVa	163514	165885	c	0.9887	2372
F084	NW_020869739.1	94167	96474	Nimav-1_LVa	45853	48192	c	0.9727	2340
F337	NW_020872253.1	768109	770465	Nimav-1_LVa	35287	37647	d	0.9970	2361
F100	NW_020869804.1	337630	339914	Nimav-1_LVa	91862	94155	d	0.9921	2294
F152	NW_020870185.1	509743	512016	Nimav-1_LVa	45866	48157	c	0.9642	2292
F063	NW_020869297.1	123798	126044	Nimav-1_LVa	260294	262561	d	0.9956	2268
F085	NW_020869739.1	96479	98687	Nimav-1_LVa	28188	30393	c	0.9804	2206
F272	NW_020871279.1	188078	190264	Nimav-1_LVa	264578	266765	d	0.9959	2188

F098	NW_020869804.1	325446	327602	Nimav-1_LVa	99697	101854	d	0.9981	2158
F163	NW_020870185.1	560434	562470	Nimav-1_LVa	3025	5071	d	0.9810	2047
F243	NW_020871127.1	416177	418202	Nimav-1_LVa	267231	269268	d	0.9877	2038
F028	NW_020868486.1	499572	501520	Nimav-1_LVa	134031	135908	c	0.9803	1878
F118	NW_020870086.1	98558	100485	Nimav-1_LVa	134031	135890	d	0.9682	1860
F354	NW_020872350.1	53135	54846	Nimav-1_LVa	195819	197657	c	0.9688	1839
F122	NW_020870086.1	151881	153655	Nimav-1_LVa	169827	171607	d	0.9804	1781
F288	NW_020871640.1	129117	130833	Nimav-1_LVa	114474	116201	d	0.9925	1728
F188	NW_020870265.1	193011	194729	Nimav-1_LVa	12244	13964	c	0.9954	1721
F355	NW_020872350.1	54849	56494	Nimav-1_LVa	191730	193403	c	0.9921	1674
F143	NW_020870185.1	452679	454279	Nimav-1_LVa	120415	122030	d	0.9944	1616
F010	NW_020868315.1	365609	367115	Nimav-1_LVa	258268	259815	d	0.9620	1548
F249	NW_020871249.1	213812	215226	Nimav-1_LVa	264482	266031	c	0.9805	1550
F309	NW_020871956.1	235938	237409	Nimav-1_LVa	24594	26070	c	0.9919	1477

F378	NW_020872607.1	443390	444773	Nimav-1_LVa	163514	164978	d	0.9813	1465
F018	NW_020868486.1	366929	368325	Nimav-1_LVa	271406	272803	d	0.9979	1398
F166	NW_020870185.1	569333	570702	Nimav-1_LVa	3154	4541	d	0.9726	1388
F089	NW_020869739.1	114806	116175	Nimav-1_LVa	76180	77545	d	0.9949	1366
F097	NW_020869804.1	324202	325445	Nimav-1_LVa	99690	100941	c	0.9936	1252
F235	NW_020871127.1	362209	363477	Nimav-1_LVa	263258	264525	c	0.9905	1268
F178	NW_020870249.1	208560	209705	Nimav-1_LVa	9961	11156	c	0.9646	1196
F320	NW_020872253.1	471979	473185	Nimav-1_LVa	35159	36369	c	0.9909	1211
F164	NW_020870185.1	562474	563596	Nimav-1_LVa	5179	6307	d	0.9858	1129
F229	NW_020870984.1	663651	664758	Nimav-1_LVa	5181	6307	c	0.9892	1127
F031	NW_020868486.1	522834	523834	Nimav-1_LVa	94439	95526	c	0.9635	1088
F318	NW_020872253.1	465447	466518	Nimav-1_LVa	28821	29898	d	0.9787	1078
F297	NW_020871791.1	133545	134532	Nimav-1_LVa	262629	263624	d	0.9859	996
F051	NW_020868730.1	91344	92321	Nimav-1_LVa	70144	71110	c	0.9714	967

F036	NW_020868486.1	539649	540475	Nimav-1_LVa	20635	21561	c	0.9678	927
F145	NW_020870185.1	473147	474060	Nimav-1_LVa	174503	175417	c	0.9891	915
F136	NW_020870086.1	309578	310465	Nimav-1_LVa	245122	246012	d	0.9966	891
F079	NW_020869739.1	73169	74027	Nimav-1_LVa	56638	57497	d	0.9814	860
F263	NW_020871249.1	476421	477274	Nimav-1_LVa	156333	157203	d	0.9814	871
F301	NW_020871791.1	164449	165311	Nimav-1_LVa	259592	260454	d	0.9919	863
F162	NW_020870185.1	559668	560426	Nimav-1_LVa	3019	3777	d	0.9685	759
F095	NW_020869804.1	315153	315885	Nimav-1_LVa	101440	102172	c	0.9891	733
F134	NW_020870086.1	302665	303355	Nimav-1_LVa	236711	237408	d	0.9913	698
F119	NW_020870086.1	101589	102169	Nimav-1_LVa	136596	137176	d	0.9556	581
F239	NW_020871127.1	379712	380292	Nimav-1_LVa	238209	238800	c	0.9585	592
F087	NW_020869739.1	101935	102489	Nimav-1_LVa	23968	24517	d	0.9586	550
F130	NW_020870086.1	263093	263658	Nimav-1_LVa	201938	202500	c	0.9510	563
F177	NW_020870249.1	207979	208542	Nimav-1_LVa	11280	11847	c	0.9912	568

F054	NW_020868730.1	136164	136697	Nimav-1_LVa	23995	24525	c	0.9664	531
F175	NW_020870249.1	197012	197539	Nimav-1_LVa	23995	24525	c	0.9755	531
F310	NW_020871956.1	237742	238274	Nimav-1_LVa	23995	24525	c	0.9813	531
F083	NW_020869739.1	93727	94166	Nimav-1_LVa	29951	30393	c	0.9751	443
F151	NW_020870185.1	509303	509742	Nimav-1_LVa	29950	30393	c	0.9729	444
F161	NW_020870185.1	559230	559667	Nimav-1_LVa	4098	4536	d	0.9500	439
F007	NW_020868315.1	338461	338881	Nimav-1_LVa	241792	242212	c	0.9667	421
F237	NW_020871127.1	376617	377035	Nimav-1_LVa	241792	242210	d	0.9689	419
F086	NW_020869739.1	98702	99159	Nimav-1_LVa	18262	18656	c	0.9597	395
F158	NW_020870185.1	553000	553385	Nimav-1_LVa	3340	3725	c	0.9742	386
F159	NW_020870185.1	553386	553767	Nimav-1_LVa	3718	4097	c	0.9738	380
F200	NW_020870398.1	133689	134063	Nimav-1_LVa	2443	2818	d	0.9515	376
F376	NW_020872607.1	433269	433609	Nimav-1_LVa	156323	156673	d	0.9710	351
F317	NW_020872166.1	152998	153269	Nimav-1_LVa	249972	250243	c	0.9816	272

F065	NW_020869429.1	159484	159711	Nimav-1_LVa	224391	224621	c	0.9652	231
F066	NW_020869429.1	173876	174103	Nimav-1_LVa	224386	224613	d	0.9605	228
F067	NW_020869429.1	192004	192231	Nimav-1_LVa	224386	224613	c	0.9868	228
F068	NW_020869429.1	235853	236078	Nimav-1_LVa	224388	224613	c	0.9779	226
F193	NW_020870319.1	581879	582104	Nimav-1_LVa	224388	224613	c	0.9646	226
F206	NW_020870618.1	55691	55915	Nimav-1_LVa	224388	224613	d	0.9867	226
F207	NW_020870618.1	90851	91076	Nimav-1_LVa	224386	224613	d	0.9824	228
F208	NW_020870618.1	99079	99301	Nimav-1_LVa	224389	224613	d	0.9866	225
F209	NW_020870618.1	174420	174647	Nimav-1_LVa	224387	224613	d	0.9561	227
F210	NW_020870618.1	180562	180786	Nimav-1_LVa	224387	224613	d	0.9867	227
F211	NW_020870618.1	268204	268431	Nimav-1_LVa	224386	224613	d	0.9825	228
F212	NW_020870618.1	268474	268698	Nimav-1_LVa	224387	224613	d	0.9823	227
F213	NW_020870618.1	283561	283793	Nimav-1_LVa	224386	224613	c	0.9696	228
F214	NW_020870618.1	290010	290234	Nimav-1_LVa	224387	224613	d	0.9823	227

F215	NW_020870618.1	329445	329667	Nimav-1_LVa	224389	224613	d	0.9777	225
F216	NW_020870618.1	390383	390608	Nimav-1_LVa	224386	224613	d	0.9824	228
F217	NW_020870618.1	390640	390867	Nimav-1_LVa	224386	224613	d	0.9825	228
F218	NW_020870618.1	390899	391126	Nimav-1_LVa	224386	224613	d	0.9825	228
F219	NW_020870618.1	391177	391402	Nimav-1_LVa	224386	224613	d	0.9780	228
F220	NW_020870618.1	413945	414164	Nimav-1_LVa	224391	224613	d	0.9820	223
F222	NW_020870618.1	489954	490178	Nimav-1_LVa	224387	224613	d	0.9823	227
F362	NW_020872505.1	2685	2911	Nimav-1_LVa	224383	224613	d	0.9696	231
F365	NW_020872505.1	280580	280799	Nimav-1_LVa	224392	224613	d	0.9774	222
F366	NW_020872505.1	321304	321527	Nimav-1_LVa	224392	224613	d	0.9820	222
F367	NW_020872505.1	330596	330814	Nimav-1_LVa	224392	224612	d	0.9864	221
F368	NW_020872505.1	490523	490743	Nimav-1_LVa	224391	224613	d	0.9820	223
F369	NW_020872505.1	503120	503340	Nimav-1_LVa	224391	224613	d	0.9820	223
F370	NW_020872505.1	524087	524305	Nimav-1_LVa	224393	224613	d	0.9773	221

F371	NW_020872505.1	584743	584960	Nimav-1_LVa	224394	224613	d	0.9863	220
F372	NW_020872515.1	300563	300789	Nimav-1_LVa	224388	224613	c	0.9559	226
F379	NW_020872607.1	528133	528358	Nimav-1_LVa	224386	224613	c	0.9782	228
